# Cultural adaptation and psychometric validation of the Health Literacy Instrument for Adults with Tuberculosis (HELIA-TB) in India

**DOI:** 10.1371/journal.pone.0352661

**Published:** 2026-06-30

**Authors:** Arohi Chauhan, Kapil Govani, Jaya Singh Kshatri, Sandeep Chauhan, Punit Patel, Joseph Baluku, Abhinav Sinha, Marjan Van Den Akker, Jesse Jansen, Jose Valderas, Sanghamitra Pati

**Affiliations:** 1 Maastricht University, Maastricht, Netherlands; 2 South Asian Institute of Health Promotion, Bhubaneswar, Odisha, India; 3 Department of Community Medicine, GMERS, Junagadh, Gujarat, India; 4 Indian Council of Medical Research- Regional Medical Research Centre, Bhubaneswar, Odisha, India; 5 Drug Resistant Tuberculosis, WHO National Tuberculosis Elimination Program Technical Support Network, New Delhi, India; 6 Department of Community Medicine, Banas Medical College and Research institute, Palanpur, Gujarat, India; 7 Makerere University Lung Institute, Kampala, Uganda; 8 Division of pulmonology, Kiruddu National Referral Hospital, Kampala, Uganda; 9 General Practice and Family Medicine, Medical School OWL, Bielefeld University, Bielefeld, Germany; 10 Department of Family Medicine, Maastricht University, Maastricht, Netherlands; 11 Maastricht University, Maastricht, Netherlands; 12 Centre for research in Health Systems Performance, Yong Loo Lin School of Medicine, National University of Singapore, Singapore, Singapore; 13 Indian Council of Medical Research, New Delhi, India; 14 ICMR-Regional Medical Research Centre, Bhubaneswar, Odisha, India; Kandahar University, Faculty of Medicine, AFGHANISTAN

## Abstract

**Background:**

Tuberculosis (TB) remains a critical public health challenge in India, where sustained patient engagement is essential for its effective management. Despite the pivotal role of health literacy, the ability to access, understand, appraise, and apply health-related information in treatment adherence and outcomes, few tools are TB specific, culturally attuned to India, and psychometrically validated. The Health Literacy Instrument for Adults (HELIA), developed in Iran, is a multidimensional, World Health Organization (WHO)-aligned measure with proven reliability and open access, making it an ideal candidate for adaptation. This study aimed to culturally adapt and validate the HELIA for use among adults receiving TB treatment in India (HELIA-TB).

**Methods:**

An exploratory sequential mixed-methods design was employed for the cultural adaptation and psychometric validation of the HELIA-TB in Junagadh district, Gujarat, India, between March 2024 and March 2025. The qualitative phase included expert review, forward translation, cognitive interviews with adults with TB and  frontline healthcare workers, back translation, and pilot testing, following international cross-cultural validation guidelines. Findings from the qualitative phase directly informed item modification, simplification of terminology, and contextual adaptation of the HELIA-TB prior to quantitative psychometric validation. The finalized instrument was subsequently administered to 393 adults with TB to assess internal consistency, test-retest reliability, content validity, and construct validity.

**Results:**

HELIA-TB retained the original five domains, access, reading, understanding, appraisal, decision-making with TB specific modifications. Internal consistency and test-retest reliability were high (α = 0.82–0.89 across domains; 0.86 overall; ICC = 0.88), and so was content validity (S-CVI/Ave = 0.92). Health literacy scores were significantly associated with treatment adherence (Cohen's d = 0.89, p < 0.001) and self-rated health (Cohen's d = 0.76, p < 0.001). No significant differences were observed by TB type or drug resistance status.

**Conclusions:**

The adapted HELIA-TB demonstrated satisfactory psychometric properties and may support assessment of health literacy and development of targeted TB care interventions in the Indian context.

## Background

Tuberculosis (TB) constitutes a persistent and complex public health challenge in India, which continues to report the world's highest incidence (199 cases per 1,00,000 population) and mortality (23 per lakh population) associated with the disease, according to the India TB Report 2024 [1]. The effective management of TB necessitates not only appropriate pharmacological interventions but also patient engagement across the entire continuum of care [2]. This includes timely recognition of symptoms, pursuit of accurate diagnosis, navigation of often fragmented health systems, adherence to extended treatment regimens, and the implementation of preventive measures to limit transmission [3]. The capacity of individuals to undertake these actions is inextricably linked to health literacy, a multifaceted construct encompassing the ability to access, comprehend, critically evaluate, and apply health information in making informed health decisions [4].

Within the context of TB care, health literacy is recognized as extending beyond basic functional skills, such as reading and understanding medication instructions [5]. It encompasses the ability to engage in meaningful dialogue with healthcare providers, assess the validity and relevance of health information, and make informed decisions that promote adherence to treatment and reduce the risk of ongoing transmission [5]. Deficits in health literacy among TB-affected populations have been correlated with suboptimal outcomes, including misinterpretation of clinical guidance, delayed healthcare-seeking behaviors, and incomplete or erratic treatment adherence [3]. Such challenges are further accentuated in resource-limited environments, where additional barriers such as poverty, social stigma, and limited access to education prevail [3].

Given the substantial impact of health literacy on TB outcomes, the development and application of valid and contextually relevant measurement instruments are of paramount importance. However, recent systematic reviews by Chauhan et al. have identified significant gaps in the availability of standardized, validated tools appropriate for use in India or similar low- and middle-income settings (LMICs) [3,6]. Specifically, nine instruments have been identified for use in TB populations and seventeen in the context of multimorbidity, yet only a limited subset have undergone psychometric evaluation in the Indian context or address the full spectrum of health literacy competencies pertinent to TB management [3,6].

Among these instruments, the Health Literacy Questionnaire (HLQ) and the European Health Literacy Survey Questionnaire (HLS-EU) have been most frequently utilized and are regarded for their comprehensive coverage of all dimensions of health literacy including functional, interactive and critical domains as well as for their robust psychometric properties [7,8]. Nevertheless, their implementation in resource-constrained contexts such as India has been circumscribed by procedural and administrative requirements, including licensing agreements and conditions governing adaptation [8]. These factors may introduce barriers to independent contextual adaptation. Other widely cited measures, such as the Rapid Estimate of Adult Literacy in Medicine (REALM) and the Test of Functional Health Literacy in Adults (TOFHLA), primarily assess functional aspects of health literacy and do not evaluate more complex skills such as critical appraisal and informed decision-making that are increasingly acknowledged as essential in TB care [9]. Additionally, some Chinese health literacy instruments have demonstrated contextual relevance for low-resource settings, yet full versions are seldom accessible in the public domain, and permissions for adaptation or translation are challenging to obtain [3,10].

While the development of new, context-specific health literacy instruments has been pursued, this approach is often resource-intensive and can limit comparability across settings. As a result, culturally adapting and validating established instruments has increasingly been recognized as a practical and scientifically efficient approach that facilitates contextual relevance while maintaining comparability across settings [11]. In this context, the Health Literacy Instrument for Adults (HELIA), developed in Iran, stands out as a promising candidate [12]. HELIA is freely available, has demonstrated robust psychometric properties in an LMIC context, and assesses multiple domains of health literacy aligned with the WHO's framework [12,13]. Thus, the objective of this study was to culturally adapt and psychometrically validate the HELIA tool for adults receiving TB treatment in India (HELIA-TB), with the aim of providing a contextually appropriate instrument to support health literacy assessment and inform targeted interventions in this high-burden setting.

## Methods

### Study design and setting

This study employed an exploratory sequential mixed‑methods design suitable for instrument adaptation and psychometric validation in a new context. The qualitative phase, comprising expert review and cognitive interviews, was undertaken first to evaluate the conceptual relevance, cultural appropriateness, linguistic clarity, and contextual suitability of the HELIA items for adults with TB. This step was necessary to ensure that the adapted items were understandable and acceptable within the local sociocultural and health‑system environment. The subsequent quantitative phase involved psychometric testing to evaluate the validity and reliability of the adapted HELIA‑TB instrument. This mixed‑methods design is well‑suited for cross‑cultural instrument adaptation, as it allows qualitative insights to inform the refinement of items prior to quantitative validation, particularly in settings where literacy levels, patient experiences, and healthcare navigation patterns differ from those in which the original tool was developed. The study was conducted between March 2024 and March 2025 in Junagadh district, Gujarat, India, and participant recruitment was conducted following approval from the Institutional Ethics Committee of GMERS Medical College, Junagadh.

HELIA was originally developed in Iran, and the English version provided by the original developers was used as the reference version during the present cultural adaptation process. It was selected as it is freely available, psychometrically validated, and designed for adult use in LMIC (classified as LMIC till 2024) setting with sociocultural and health system parallels to India [12]. The tool assesses five domains aligned with the WHO definition of health literacy, including functional, interactive, and critical domains, i.e., access to information, reading, understanding, appraisal, and decision-making [12]. For this study, the instrument was adapted for two aspects: (1) tailoring the content to the TB and (2) translating and culturally adapting the language for the Gujarat context.

Junagadh district, located in the Saurashtra region of Gujarat state, includes urban, rural, and tribal communities. The district is characterized by an overall literacy rate of approximately 76%, but contains sizable populations with low literacy [14]. As part of Gujarat, which has a high burden of TB and coexisting comorbidities, Junagadh is served by the National TB Elimination Program (NTEP) through a network of public and private health facilities [1].

### Study participants

The adaptation and validation of the HELIA-TB tool engaged multiple participant groups at distinct stages. The initial expert review purposively selected three professionals representing public health research, TB clinical expertise, and TB program management. Their ages ranged from 38 to 48 years, with 10–25 years of professional experience in TB care and public health. These experts provided input on the contextual adaptation of the instrument. During cognitive interviews, eight healthcare professionals selected through snowball sampling contributed insights on the tool’s clarity and field relevance. This group included Accredited Social Health Activists (ASHAs), medical officers, state TB supervisors (treatment and laboratory), and a DOT provider, reflecting community, clinical, and supervisory perspectives. Participants ranged in age from 25 to 48 years and had between 8 and 20 years of work experience in TB services and public health practice. The expert panel for content validity consisted of another eight subject-matter experts identified through snowball sampling, representing program management, development partners, research, academia, and public health practice. Their ages ranged from 36 to 48 years, with 12–27 years of professional experience, ensuring multidisciplinary review of the adapted instrument. Details of experts and healthcare providers are presented in S1 Table.

Five adults with TB purposively selected with diverse demographic and clinical characteristics participated in the cognitive interviewing phase, while thirty adults with TB participated in the pilot testing phase. Among pilot participants, the mean age was 41.3 years (SD ± 10.2; range 19–65 years), 47% were female, 20% had no formal schooling, and 22% reported at least one comorbid condition such as diabetes, HIV, or hypertension. Participants were recruited from both public and private healthcare settings and represented varying literacy and treatment backgrounds. Further, internal consistency and construct validity were evaluated using a larger sample of 393 adults with TB.

### Adaptation

The adaptation process followed internationally accepted standards for cross-cultural adaptation and validation, drawing on frameworks from the Human Services Research Institute (HSRI) [15].

**Fig 1:**
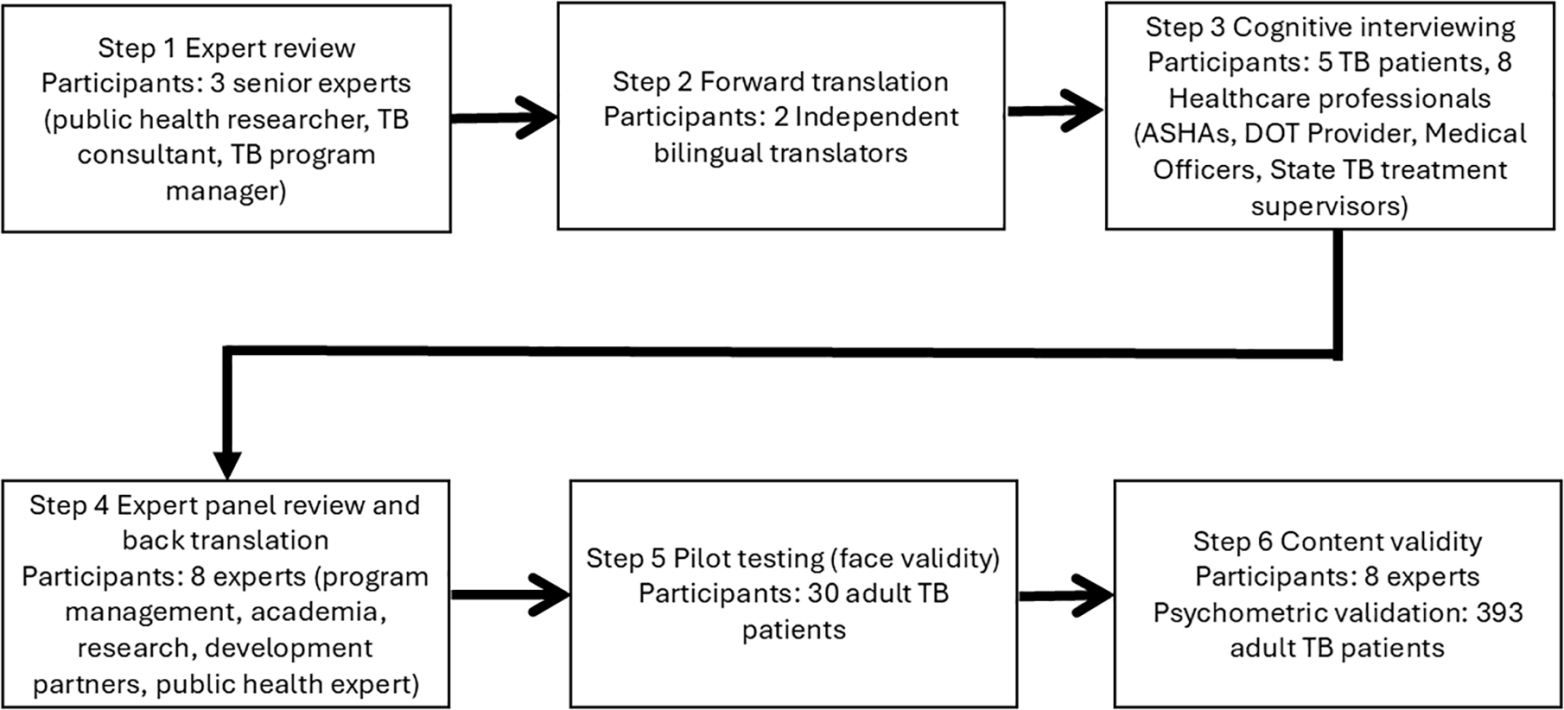
Steps of adaptation and validation of HELIA-TB and participants involved.

The process included the following phases (Fig 1):

#### Expert review and cultural relevance assessment.

The original English version of the HELIA questionnaire was reviewed by a panel of three senior experts in public health and TB care. Each item was evaluated for conceptual relevance, clarity, and sensitivity within the Indian context of TB and multimorbidity.

#### Forward translation.

The adapted tool was forward translated from English into Gujarati by two independent bilingual translators. Emphasis was placed on achieving semantic, idiomatic, and conceptual equivalence, rather than literal translation.

#### Cognitive interviewing.

The Gujarati version was tested via semi-structured cognitive interviews with a purposive sample of five adults with TB and eight frontline health workers (S1 Table). These interviews explored participant comprehension, emotional acceptability, cultural relevance, and clarity of each item. Although the number of patient interviews was limited due to feasibility constraints, the discussions were in-depth and provided valuable insights. To supplement this, eight frontline health workers, who play a central role in TB education and communication, particularly in low-literacy settings, were included to provide complementary perspectives based on their extensive patient interactions. Feedback from both groups informed revisions to terminology, simplification of complex phrases, and improved sensitivity to stigma and literacy challenges. While the principle of data saturation guided this phase, the cognitive testing was further strengthened by subsequent large-scale field testing and psychometric validation with 393 adults with TB.

#### Expert panel review and back translation.

An expanded panel of separate eight experts, including stakeholders from TB programs, academia, research, development partners, and public health practice, reviewed the revised Gujarati version (S1 Table). Items were evaluated for relevance, clarity, simplicity, and cultural appropriateness. The tool was then back-translated into English by an independent translator to ensure conceptual fidelity. The back translator was not involved in the original translation process and was blinded to the original English version to ensure objectivity.

#### Field testing.

The finalized tool was prepared for field testing to assess its psychometric properties among 30 adults with TB selected to ensure demographic and clinical diversity. This pilot sample was used to evaluate feasibility, acceptability, and inform formal validation measures such as content validity and test–retest reliability.

#### Validation and reliability testing.

**Face validity.** Face validity of the adapted HELIA-TB was assessed through pilot testing with 30 adults with TB selected to ensure diversity in gender, age, treatment settings (public and private), literacy level, and comorbidity status. The assessment aimed to ensure clarity, practicality, and acceptability among adults with TB. Feedback was gathered through patient debriefing, structured response forms, and qualitative feedback, following best practices for community-based tool adaptation. Field workers, including ASHAs, treatment providers, and supervisors, also provided input as future administrators of the tool. They completed structured questionnaires with Likert-scale items assessing clarity, relevance, practicality, and patient comfort, along with open-ended sections for further suggestions. Additionally, focus group discussions (FGDs) were held with field workers to explore the appropriateness of individual items, any potential discomfort, and the feasibility of tool use in low-resource settings. Insights from these multiple sources informed final refinements to enhance the tool’s usability and acceptability.

**Internal Consistency.** Reliability of the tool was assessed using Cronbach’s alpha, calculated for each domain and the overall scale to measure internal consistency.

**Test-retest Reliability.** To evaluate temporal stability, the tool was re-administered to a subset of 30 participants after a 30-day interval. Intraclass Correlation Coefficients (ICCs) were calculated for each domain and the overall scale.

**Content Validity.** Content validity of the HELIA-TB was established in a two-phase process conducted after initial face validation. This stage was designed to assess whether the tool adequately captures relevant domains of health literacy for adults with TB, using both qualitative and quantitative approaches guided by recognized validation frameworks. The total health literacy scores (HELIA-TB) were also analyzed for distribution and potential clustering at the extreme ends.

### Sample size and sampling

Sample sizes for each phase were informed by methodological standards for cross-cultural adaptation and psychometric validation. For pilot testing, a sample of 30 adults with TB was selected based on the commonly applied rule of thumb that 20–30 participants are sufficient to assess clarity, feasibility, and preliminary reliability before large-scale validation. The HELIA-TB instrument consisted of 33 items across five domains. For the validation phase, 393 adults with TB were included, exceeding the minimum recommended ratio of 5–10 participants per item for factor-analytic procedures and reliability testing, with approximately 12 participants per item, thereby ensuring sufficient statistical power and representativeness. For Confirmatory Factor Analysis (CFA), the validation sample size was justified based on model complexity, estimation requirements, and commonly recommended minimum sample thresholds for structural equation modeling. The final sample of 393 participants was considered adequate for maximum likelihood CFA because it exceeded commonly suggested minimum sample sizes of 200–300 cases for stable parameter estimation and provided sufficient observations relative to the 33-item, five-factor measurement model. This sample size also allowed estimation of factor loadings, inter-factor correlations, and model fit indices with acceptable precision.

### Sampling method

For the pilot testing and validation phases, participants were randomly selected from TB treatment registers maintained at District TB centers under the NTEP. Eligible participants included adults with TB currently receiving treatment during the study period. Simple random sampling was applied within the available treatment registers to minimize selection bias and improve representativeness across treatment settings. Recruitment was conducted during routine outpatient visits, resulting in a high participation rate (>95%) and minimal non-response

### Description of measures

**HELIA-TB.** The adapted HELIA-TB included five domains aligned with the WHO definition of health literacy: functional, interactive, and critical, covering access to information, reading, understanding, appraisal, and decision-making.

**Content Validity Assessment**: A structured expert review was conducted using a content validation questionnaire. Experts rated each item on a 4-point Likert scale for relevance (1 = Not Relevant to 4 = Highly Relevant) and a 5-point scale for clarity (1 = Not Clear to 5 = Very Clear) and simplicity (1 = Complex to 5 = Very Simple). Additional criteria included cultural sensitivity and comprehensiveness. Experts could also provide qualitative feedback via open-text comments.

**Treatment Adherence**: Data were extracted from patient treatment report cards maintained at participating health facilities under the NTEP. Adherence was classified as regular (≥80% doses taken within 120% of expected treatment duration) or irregular (<80% doses taken within 120% of duration), based on the most recent treatment period prior to HELIA-TB administration.

**Self-Rated Health**: Self-rated health was measured using the Multi-morbidity Assessment Questionnaire for Primary Care (MAQ-PC), validated in the Indian setting [2]. Participants rated their current health on a 5-point Likert scale from “very poor” to “very good.”

### Data analysis

Data analysis was conducted in two sequential phases, integrating qualitative and quantitative methodologies. In the cultural adaptation phase, qualitative data from expert review forms and cognitive interview notes were thematically analyzed to evaluate item clarity, cultural relevance, and emotional acceptability. In the validation phase, quantitative analyses were performed using SPSS version 26.0. HELIA-TB scores were linearly transformed to a standardized 0–100 scale according to the original HELIA scoring protocol, with higher scores indicating higher health literacy levels. CFA was conducted using the IBM SPSS AMOS version 26.0 with maximum likelihood estimation to test the fit of the original five-domain structure of HELIA-TB. Content validity was assessed by calculating the Item-Level Content Validity Index (I-CVI) and Scale-Level Content Validity Index (S-CVI) from expert ratings obtained through structured content validation questionnaires. Internal consistency reliability was examined using Cronbach’s alpha for each domain and for the overall scale. Score distribution was analyzed for skewness, kurtosis, and range to detect potential floor and ceiling effects. Test-retest reliability was determined by calculating the ICC from a subset of participants who completed the tool twice, 30 days apart. Descriptive statistics were used to summarize participant demographics and responses related to tool acceptability and feasibility.

Construct validity was evaluated using a larger, independent sample of 393 adults with TB. Predictive validity was assessed by comparing HELIA-TB scores between regular and irregular adherence groups using independent t-tests. Convergent validity was examined by correlating health literacy scores with self-rated health status, while discriminant validity was determined by analyzing score differences according to TB type (pulmonary vs. extrapulmonary) and drug resistance status (drug-sensitive vs. drug-resistant). Additionally, subgroup analyses by educational attainment were performed to assess tool performance among participants with lower literacy levels. CFA was conducted using maximum likelihood estimation to evaluate the fit of the original five-domain structure of the HELIA-TB instrument. Preliminary assessment of data distribution included examination of skewness, kurtosis, and inter-item correlations to assess the suitability of the estimation method. Model fit was evaluated using multiple fit indices, including the chi-square statistic (χ²), χ²/df ratio, Comparative Fit Index (CFI), Tucker-Lewis Index (TLI), Root Mean Square Error of Approximation (RMSEA), and Standardized Root Mean Square Residual (SRMR). Modification indices and correlated residuals between conceptually related items were reviewed during model refinement while preserving the theoretical structure of the original HELIA framework. Convergent validity was assessed using Composite Reliability (CR) and Average Variance Extracted (AVE), while discriminant validity was examined through inter-factor correlations and comparison of AVE estimates across latent constructs.

### Ethics considerations and consent to participate

This study was approved by the Institutional Ethics Committee of GMERS Medical College, Junagadh (IEC/99/2024). The study was conducted in accordance with the principles of the Declaration of Helsinki. Informed verbal consent was obtained from all adult participants prior to data collection and was documented by the interviewer in accordance with the approved protocol. No minors were included in the study. Confidentiality and anonymity were strictly maintained throughout the study.

### Consent to publication

All authors approved the final manuscript and consented to its publication.

## Results

### Contextualization and adaptation of the tool

The process began with a review by three senior public health experts, who evaluated the original English HELIA items for their applicability to the TB and co-morbidity context. Based on this review:

- Item 6 (“I can find health information about healthy eating”) was replaced with:

*“I can find health information about TB.”*

- Item 25 (“If anyone from my first-degree relatives develops cancer... I see a doctor to examine me”) was replaced with:

*“If anyone in my family develops cough or weight loss, I see a doctor to examine them.”*

- Item 30 (“I buy dairy products according to their fat percentage”) was contextually modified to:

*“I buy food products (like oil or milk) after checking if they are healthy for someone with TB.”*

These adaptations were made to reflect real-life behaviors, and information needs specific to TB care, family symptom recognition, and nutrition choices. Each revised or modified item was assessed for its alignment with the original HELIA domain, access to information, decision-making, and appraisal, respectively. These changes preserved the original intent and structure of the domains. Formal approval for these modifications was sought from the original developer of HELIA, who confirmed that the revised items remained aligned with their respective domains. Since no constructs were altered during adaptation, the developer approved the changes and shared the scoring sheet for use with the adapted version.

### Forward translation and cognitive interview

Following forward translation by two independent translators, cognitive interviews were conducted with five adults with TB and eight healthcare providers (S1 Table). Participants suggested synonyms for difficult or technical terms, highlighted specific cultural barriers, and confirmed the importance of including decision-making and health system navigation aspects. This phase ensured that the tool was understandable, locally relevant, and did not trigger negative emotions for adults with TB. Around seven changes were made including revising terminology from “physician” to “doctor”, “high blood sugar” to “diabetes”, “high blood pressure” to “BP problem”, “TB” to “health condition”, “admission, consent forms, etc.” to “hospital forms”, “radiology” to “x-ray”, and “signage” to “signs/boards in hospital”.

### Expert panel review and back translation

The final instrument retained all five core domains of the original HELIA tool, reading, access, understanding, appraisal, and decision-making/behavior, while incorporating TB specific terminology and content reflective of multimorbidity realities. Based on expert panel feedback, minor revisions were made to enhance contextual relevance; however, no items were removed or entirely replaced. This adapted version was considered ready for psychometric validation, which was subsequently conducted through a cross-sectional study involving adults with TB (S2 Table).

### Face validity

The finalized, contextualized version of the HELIA-TB was pilot-tested among 30 adults with TB currently undergoing treatment at both public and private health facilities. The mean age was 41.3 years (SD ± 10.2; range 19–65), and 47% of participants were female. The majority (63%) received care in public sector facilities, with the remainder attending private clinics. Time since diagnosis ranged from 1 to 8 months. Regarding educational status, 20% of patients reported no formal schooling, while 80% had at least some formal education. Additionally, 22% of participants reported at least one comorbid condition, such as diabetes, HIV, or hypertension. Over 95% reported that the questions were easy to understand, contextually relevant, and non-threatening. No items were flagged as confusing, stigmatizing, or intrusive. This level of acceptability confirmed that the instrument was linguistically and emotionally accessible, i.e., did not trigger negative emotions, to a diverse patient population, including individuals with limited literacy and those living with TB multimorbidity.

Further, most field staff rated the tool highly on simplicity, time efficiency, and practical applicability in real-world community settings. FGDs with the field workers noted that the tool was of an appropriate length, required no special training, and could easily be integrated into routine patient interaction workflows. Follow-up one-on-one interviews were also conducted with a subset of participants from both groups to triangulate responses and gather more nuanced suggestions for improvement. The collective feedback from these validation exercises guided minor modifications in phrasing and affirmed that the tool was well-suited for deployment among adults with TB in both clinical and community settings.

### Content validity indices

Based on expert ratings, the adapted HELIA-TB demonstrated strong content validity [16]. The S-CVI, calculated as the average of all I-CVIs across the scale, was 0.92, indicating excellent overall agreement among experts. The I-CVI was calculated as the proportion of experts rating an item as either “quite relevant” or “highly relevant.” I-CVI values ranged from 0.88 to 1.00, indicating high agreement among experts regarding item relevance. These quantitative results confirm that the adapted items were viewed as highly relevant, conceptually appropriate, and linguistically clear for the intended population. In addition, experts rated most items highly for clarity and simplicity, with the majority receiving scores of 4 or 5 on the 5-point Likert scales. Qualitative feedback further supported these findings, with experts affirming the tool’s appropriateness for low-literacy populations and its cultural sensitivity (Table 1). Although modified kappa coefficients were not calculated to adjust for chance agreement, the consistently high I-CVI and S-CVI/Ave values suggested strong overall expert consensus.

**Table 1:** Content validity and expert evaluation of HELIA-TB items.

Item No.	Item Description	I-CVI	Clarity Mean (1–5)	Simplicity Mean (1–5)	Expert Comments
1	Reading educational materials about TB (booklet, pamphlets, posters) is easy for me.	1.00	4.9	4.8	Very relevant; suggested adding local examples like TB posters in clinics
2	Reading written instructions from doctor and health workers about TB is easy for me.	0.88	4.5	4.6	Add ‘in simple language’ to improve clarity for low-literacy patients
3	Reading medical forms (such as admissions, consent, filing, etc. in hospitals and medical centers) is easy for me.	0.88	4.3	4.4	Clarify terms like ‘symptoms’ or ‘diagnosis’
4	Reading pamphlets and instructions for laboratory testing, sputum testing, ultrasound or radiology is easy for me.	1.00	4.7	4.8	No revisions needed
5	I can find health information from different sources when I need such information.	1.00	5.0	4.9	Clear and practical
6	I can find health information about TB.	0.88	4.5	4.6	Some patients might confuse ‘information’ with instructions. Add ‘in simple language’ for clarity
7	I can find health information on mental health such as depression and stress.	0.88	4.4	4.5	Suggest rephrasing to ‘I feel confident to ask questions’
8	I can find health information about a specific disease when I need to.	1.00	4.6	4.7	Useful for encouraging family communication
9	I can find health information for some health problems and diseases such as high blood pressure, diabetes and high lipid levels.	0.88	4.2	4.3	Consider including psychological or mental health symptoms
10	I can find health information about harmful effects of tobacco and smoking.	0.88	4.3	4.2	Recommend using the phrase “true or correct” in translation
11	I can understand the recommendations for a healthy diet.	0.88	4.4	4.5	Suggest using examples relevant to TB (e.g., protein-rich foods)
12	I can understand when my doctor explains about my illness (TB).	1.00	4.8	4.7	Clear and important for treatment adherence
13	I can understand the meaning when reading medical forms in hospitals and health centers.	0.88	4.3	4.3	Use simple language; may be difficult for low-literacy groups
14	I can understand signs or boards in hospitals, clinics and health centers.	0.88	4.5	4.6	Good item; suggest adding local signage examples
15	I can understand drug information on labels.	1.00	4.7	4.7	Clear and appropriate
16	I can understand the risks and benefits of drugs prescribed by my doctor.	0.88	4.3	4.4	Could add ‘explained in simple terms’
17	I can understand written information before sputum testing, ultrasound or radiology.	0.88	4.2	4.3	Good for informed consent; include examples like chest X-ray
18	I can evaluate health-related information on the Internet.	0.88	4.1	4.0	Less relevant for low-resource settings; still useful for urban adults with TB
19	I can evaluate health-related information broadcast on television and radio.	1.00	4.5	4.6	Important for mass awareness; suggest referencing TB ads
20	I can assess the accuracy of health-related recommendations I receive from relatives and friends.	0.88	4.3	4.4	Clear and relevant
21	I can communicate trusted health information to others.	1.00	4.5	4.6	Encourages patient empowerment
22	When facing an illness, I know where to go or with whom to speak.	1.00	4.7	4.8	Crucial for timely treatment-seeking behavior
23	When doctor suggests that I should take medicines three times a day, I know that I should take one tablet every 8 hours.	1.00	4.9	4.8	Very clear; supports treatment adherence
24	I do not cut my medications without my doctor’s permission, even if symptoms disappear.	1.00	5.0	4.9	Strong item; good for adherence messaging
25	If anyone in my family develops cough or weight loss, I see a doctor to examine them.	0.88	4.6	4.5	Adapted from cancer item to TB specific symptoms
26	I avoid doing or eating things that increase my blood pressure.	0.88	4.3	4.3	Relevant for comorbidity (hypertension); suggest TB context if retained
27	I visit my doctor for regular checkups.	1.00	4.8	4.8	Simple and strongly relevant
28	I am health-conscious in any situation.	0.88	4.2	4.2	Broad statement; consider specific examples
29	I ask my doctor or health care staff questions about my disease when needed.	1.00	4.7	4.6	Reinforces patient participation
30	I buy food products (like oil or milk) after checking if they are healthy for someone with TB.	0.88	4.4	4.3	Modified from fat-content item; relevant for nutrition guidance, prevention of multimorbidity
31	I ensure to take substances that increase my weight.	0.88	4.2	4.3	Simple and relevant
32	I use a helmet or seat belt while driving.	0.88	4.1	4.2	Relevant, multimorbidity
33	I consider the food labels when shopping.	0.88	4.3	4.4	Supports nutritional awareness

### Feedback-driven revisions and final expert consensus

In addition to numerical ratings, expert suggestions were used to refine item language and structure. Several terms were rephrased to enhance accessibility for low-literacy populations, and minor adjustments were made to ensure cultural sensitivity. No items were flagged for removal, and no additional items were recommended, confirming that the tool was seen as comprehensive yet concise. Following these refinements, a final version of the HELIA-TB was shared with the same expert panel to confirm that the revised items adequately reflected their feedback. All experts expressed agreement that the adapted version retained the construct integrity of the original HELIA while being fully relevant and usable within the TB care context.

### Test-retest reliability

The adapted HELIA-TB demonstrated excellent test–retest reliability, with an ICC [2,1] of 0.88 for the total scale score. This high ICC value reflects strong temporal stability of the instrument, indicating that respondents provided consistent answers when the tool was administered twice, 30 days apart (Table 2).

**Table 2:** Reliability statistics for the HELIA-TB tool.

Domain	No. of Items	Cronbach’s Alpha	ICC (95% CI)	Interpretation
Access to Information	6	0.82	0.87 (0.75–0.94)	Excellent
Reading	4	0.84	0.89 (0.78–0.95)	Excellent
Understanding	7	0.86	0.88 (0.76–0.94)	Excellent
Appraisal	4	0.83	0.85 (0.71–0.92)	Good to Excellent
Decision-making	12	0.89	0.90 (0.80–0.96)	Excellent
Overall Scale	33	0.86	0.88 (0.79–0.95)	Excellent

Note: ICC = Intraclass Correlation Coefficient; CI = Confidence Interval. Interpretation based on commonly accepted benchmarks: ICC ≥ 0.75 = excellent; 0.60–0.74 = good; 0.40–0.59 = moderate.

### Predictive validity

Analysis of predictive validity showed that patients with good treatment adherence (n = 362) had a substantially higher mean health literacy score (M = 43.24, SD = 18.67) compared to those with poor adherence (n = 31; M = 26.80, SD = 16.81). This difference was statistically significant (p < .001), with a mean difference of 16.44 (95% CI [9.62, 23.26]) and a large effect size (Cohen’s d = 0.887, 95% CI [0.514, 1.258]). Additionally, higher health literacy scores were significantly associated with better TB treatment adherence (Table 3).

**Table 3:** Construct validity analyses of HELIA-TB tool.

Type	Variable	Group 1 (n, Mean ± SD)	Group 2 (n, Mean ± SD)	t/ p-value	Cohen’s d	Interpretation
Convergent	Self-Rated Health (SRH)	Good (348, 43.55 ± 18.58)	Poor (45, 29.52 ± 18.14)	*t*(391)=4.78, *p*<.001	0.76	Supports convergent validity
Discriminant	TB Type	Pulmonary (353, 41.32 ± 18.34)	Extra-pulmonary (40, 47.43 ± 23.91)	*t*(391)= -1.93, *p*=.054	−0.32	No significant difference
Discriminant	Drug Resistance	Sensitive (382, 42.12 ± 19.04)	Resistant (11, 35.85 ± 18.75)	*t*(391)=1.08, *p*=.283	0.33	No significant difference
Predictive	Treatment Adherence	Good (362, 43.24 ± 18.67)	Poor (31, 26.80 ± 16.81)	*t*(391)=4.74, *p*<.001	0.89	Supports predictive validity

### Convergent validity

For convergent validity (n = 393), it was hypothesized that participants reporting better overall health would demonstrate higher health literacy scores. The results supported this hypothesis: participants with positive self-rated health (n = 348) had a mean score of 43.55 (SD = 18.58), whereas those with negative self-rated health (n = 45) had a lower mean score of 29.52 (SD = 18.14). This difference was statistically significant (t(391) = 4.78, p < .001), with a mean difference of 14.03 (95% CI [8.26, 19.80]) and a moderate to large effect size (Cohen’s d = 0.757). These results further support the convergent validity of the HELIA-TB instrument (Table 3).

### Discriminant validity

By TB Type: No significant difference in health literacy scores was observed between participants with pulmonary TB (n = 353; M = 41.32, SD = 18.34) and those with extrapulmonary TB (n = 40; M = 47.43, SD = 23.91), with t(391) = −1.93, p = 0.054, and a small effect size (Cohen’s d = −0.322). This non-significant difference supports discriminant validity, indicating that clinical TB type does not influence health literacy scores (Table 3).

By Drug Resistance Status: Similarly, no statistically significant difference in health literacy was observed between those with drug-sensitive TB (n = 382; M = 42.12, SD = 19.04) and drug-resistant TB (n = 11; M = 35.85, SD = 18.75), with t(391) = 1.08, p = 0.283, 95% CI for mean difference [−5.18, 17.71], and a small effect size (Cohen’s d = 0.329). These results further confirm discriminant validity, as drug resistance status was not associated with health literacy levels (Table 3).

### Subgroup analysis by literacy level

To evaluate the performance and internal consistency of the HELIA-TB instrument across literacy strata, a subgroup analysis was conducted based on participants’ educational attainment. Reliability was assessed separately for individuals with no formal education and those with at least some formal education (Table 4).

**Table 4:** Sub-group analyses of internal consistency by education levels.

Education Level	N	Cronbach’s Alpha (Raw)	Cronbach’s Alpha (Standardized)	Interpretation
No formal education	225	0.848	0.859	Good internal consistency
Formal education (any)	168	0.904	0.919	Excellent internal consistency

Among participants with formal education (n = 168), the HELIA-TB tool demonstrated excellent internal consistency, with a Cronbach’s alpha of 0.904 (standardized α = 0.919). Inter-item correlations were moderate to strong across domains, particularly between understanding and decision-making (r = 0.787), and between reading and understanding (r = 0.749). All domains showed strong item-total correlations, with alpha values remaining high even when any individual domain was removed (range: 0.876–0.924). This suggests stable and cohesive measurement of health literacy within this group.

Among participants with no formal education (n = 225), internal consistency remained high, with a Cronbach’s alpha of 0.848 (standardized α = 0.859). Although the average inter-domain correlations were generally lower than those in the educated group, the tool still demonstrated acceptable reliability. Notably, understanding and decision-making maintained a strong correlation (r = 0.817), and item-total correlations remained moderate to strong for most domains.

### CFA

CFA was conducted to test the fit of the original five-domain structure of the HELIA-TB instrument among 393 adults with TB. The model demonstrated excellent fit (CFI = 0.946, TLI = 0.937, RMSEA = 0.051 (90% CI: 0.045–0.058), SRMR = 0.041, χ²/df = 2.41), indicating that the hypothesized structure aligned well with the observed data. All items loaded significantly onto their respective latent constructs, with standardized factor loadings ranging from 0.62 to 0.88, except for one marginal item (Q32, λ = 0.43). However, Q32 was retained because of its conceptual relevance to preventive health decision-making behaviors and its minimal impact on overall scale reliability and construct coherence. Reliability estimates across domains were strong, with composite reliability (CR) values between 0.85 and 0.92, and average variance extracted (AVE) ranging from 0.53 to 0.72. Inter-factor correlations (e.g., Understanding with Appraisal: r = 0.78; Understanding with decision-making: r = 0.82) supported the theoretical coherence of the tool’s domain structure. These results confirm the structural validity of the adapted HELIA-TB in the Indian TB care context. Inter-factor correlations remained within theoretically acceptable limits and, together with the AVE estimates, supported adequate discriminant validity between the latent domains of the HELIA-TB instrument.

### Response distribution and variability

Among 393 valid cases, the mean score was 41.94 (SD = 19.04), and the median was 40.60, indicating a slightly right-skewed but generally balanced distribution (Skewness = 0.578). Quartile analysis revealed that 25% of participants scored below 28.07 and 25% scored above 53.10, suggesting adequate spread across the scale. No significant floor or ceiling effects were observed, supporting the tool’s capacity to differentiate between varying levels of health literacy.

### Item-total correlation analysis

Item-total correlation analysis was conducted to assess the internal coherence of the HELIA-TB tool. The corrected item-total correlations ranged from 0.067 to 0.776, with most items exceeding the acceptable threshold of 0.30, indicating strong contribution to the overall construct. The overall Cronbach’s alpha was 0.86, confirming excellent internal consistency. A few items (e.g., Q32, Q20, Q31) demonstrated relatively low item-total correlations. In particular, Q32 showed a notably weak correlation (0.067) and its removal would slightly increase the alpha, suggesting a potential lack of alignment with the rest of the scale. However, given the overall high reliability and theoretical relevance of the items, all were retained for further analysis.

Inter-item correlation analysis was conducted to evaluate the internal structure of the adapted scale. The mean inter-item correlation was 0.359, which is within the optimal range (0.20–0.40) recommended for scales assessing related but distinct constructs. This suggests an acceptable level of internal consistency, with items demonstrating conceptual alignment without excessive redundancy.

## Discussion

This study provides evidence for the successful cultural adaptation and validation of the HELIA-TB, a multidimensional health literacy assessment tool for adults with TB in India. The adapted instrument demonstrated good internal consistency, excellent test-retest reliability, and robust predictive, convergent, and discriminant validity. Notably, it maintained high reliability and coherence among both literate and low-literacy participants. Higher health literacy scores were associated with better treatment adherence and favorable self-rated health, affirming the clinical importance of health literacy assessment in TB management.

These results align with international evidence on the critical role of health literacy in chronic disease outcomes [4,17]. Previous studies have consistently shown that higher health literacy is associated with improved treatment adherence, greater patient engagement, and better health status across disease contexts [18,19]. Our finding that the HELIA-TB distinguishes between patients with regular and irregular adherence reinforces evidence from both high- and low-income settings, where inadequate health literacy often correlates with missed doses, treatment interruptions, and delayed health-seeking behavior [20]. The absence of significant differences in health literacy by TB type or drug resistance status further supports the instrument’s discriminant validity and suggests that it captures fundamental capabilities relevant to all adults with TB, regardless of clinical profile.

A key strength of this work lies in its inclusive and systematic adaptation process. Engagement of patients, field workers, and experts at each stage of adaptation, translation, and validation ensured that the final tool was not only linguistically accurate but also culturally relevant and did not trigger negative emotions. The robust performance of the instrument among individuals with no formal education addresses a persistent gap in health literacy measurement, as tools are often less accessible and less valid in low-literacy populations. By demonstrating reliability and validity in this subgroup, HELIA-TB emerges as an equity-focused instrument, capable of identifying and addressing literacy-related barriers in the populations most vulnerable to TB. This equity focus is particularly important for TB elimination, as low literacy is a known driver of delayed diagnosis, poor adherence, and worse outcomes [21].

The tool’s practical applicability further enhances its value. Field staff rated it as simple, brief, and compatible with routine workflows, while over 95% of patients in pilot testing found it clear and easy to understand. These characteristics support its scalability and integration into TB programs targeting literacy-related barriers to care. Psychometrically, the adapted HELIA-TB matched or exceeded the performance of the original HELIA in Iran (original α range: 0.72–0.89; current α range: 0.82–0.89) and demonstrated higher test-retest reliability (ICC = 0.88 vs. 0.72–0.85). Content validity (S-CVI/Ave = 0.92) was also consistent with, other adapted health literacy tools, such as the Chinese TB Health Literacy Scale (S-CVI/Ave = 0.87). [10] The predictive validity of the HELIA-TB for treatment adherence (Cohen’s d = 0.89) was notably higher than the average effect sizes reported in meta-analyses of the health literacy-adherence relationship across various chronic diseases, which typically range from 0.16 to 0.60, although such comparisons should be interpreted cautiously given differences in instruments, adherence measures, and clinical contexts [19,22]. This suggests that health literacy may play a crucial role in TB treatment adherence, especially given the regimen’s length, complexity, and the frequent coexistence of other chronic conditions that can increase treatment burden. This reinforces the value of employing a TB specific, culturally adapted instrument to identify patients at risk of poor adherence and to design targeted, health literacy-sensitive interventions.

Rather than developing a new tool, this study maximized methodological rigor and resource efficiency by culturally adapting a validated instrument. This approach ensures both local relevance and global comparability, especially valuable in resource-constrained settings. The systematic process, combining cognitive interviews, expert consensus, and psychometric validation, offers a replicable model for other health domains. Importantly, innovation lies not only in content and context but in creating an inclusive, scalable framework for localizing global tools across diverse health challenges.

Despite these strengths, some limitations warrant consideration. One notable limitation of the expert review phase was the absence of direct patient involvement in the initial cultural adaptation. This sequencing was intentional, as early drafts required technical and conceptual refinement by domain experts before being shared with patients to avoid confusion or exposing them to items that had not yet been linguistically or culturally clarified. While expert feedback ensured clinical and conceptual relevance, future phases will benefit from incorporating patient perspectives to enhance contextual and experiential validity. Additionally, although Rasch analysis was explored as a complementary psychometric method, it could not be meaningfully applied because several response categories of the 5-point Likert scale showed sparse or zero frequencies particularly the extreme categories (“1 = Not at all” and “5 = Always/ Very much”), which violated key model assumptions and led to unstable parameter estimates. This reflects a methodological constraint rather than a shortcoming of the HELIA-TB tool itself. Advanced discriminant validity assessment methods such as the Fornell–Larcker criterion and Heterotrait–Monotrait (HTMT) ratio analysis were not performed in the present study. Moreover, validation was conducted in a single district using the Gujarati language, which may limit the generalizability of findings to other linguistic or regional contexts within India. However, Gujarat’s TB indicators and health system characteristics are broadly comparable to national averages, supporting the relevance of these findings. The 30-day test-retest interval may have allowed genuine changes in health literacy due to ongoing TB treatment and counseling. The small number of participants with drug-resistant TB reduced statistical power for subgroup analyses, and predictive validity analyses did not adjust for potential confounding variables. Convergent validity relied on a single-item self-rated health measure, and formal measurement invariance testing using multi-group CFA was not performed. As responses were self-reported, social desirability bias cannot be excluded. Finally, the cross-sectional design limited assessment of responsiveness, longitudinal changes, and long-term predictive utility. Future research should evaluate the HELIA-TB across multiple Indian languages and settings and examine responsiveness through longitudinal studies.

## Conclusion

The adapted HELIA-TB is a reliable, valid, and contextually appropriate instrument for assessing health literacy among adults with TB in India, including those with limited education. Its adoption in TB programs may facilitate the identification of health literacy barriers, the design of targeted education and support, and the promotion of patient-centered care to improve TB outcomes. Future research should focus on broader implementation, continued evaluation in varied settings, and exploration of the tool’s potential for driving improvements in both individual and programmatic TB management. As a novel contribution, HELIA-TB offers not only contextual adaptation but also a scalable, equity driven framework for contextualizing global tools to local disease settings, a framework with broad applicability across health systems.

### Way forward

Future research should prioritize the translation and psychometric validation of the HELIA-TB across multiple Indian languages and diverse epidemiological contexts to enhance generalizability. Longitudinal studies are warranted to evaluate its responsiveness to targeted interventions and its utility as both a screening and outcome assessment tool. Integration into national TB programs could facilitate systematic identification of patients at elevated risk for non-adherence, particularly those with multimorbidity or socio-economic disadvantage, enabling tailored, health literacy sensitive interventions. Embedding the HELIA-TB within digital health platforms and programmatic monitoring frameworks may further strengthen patient-centered TB care.

## Supporting information

S1 Table Characteristics of experts and healthcare providers involved in adaptation and validation. (DOCX)

S2 Table Health Literacy Instrument for Adults (HELIA) – TB (Adapted Version for Research Use Only). (DOCX)
