# Supplementary material for: Cultural adaptation and psychometric validation of the Health Literacy Instrument for Adults with Tuberculosis (HELIA-TB) in India
Source: PLoS One. 2026 Jun 30;21(6):e0352661. doi: 10.1371/journal.pone.0352661 (PMC13318012; doi:10.1371/journal.pone.0352661)
Supplement: S1 Table — (DOCX) [file pone.0352661.s001.docx]

S1 Table. Characteristics of Experts and Healthcare Providers Involved in Adaptation and Validation

| **Group** | **Age Group (years)** | **Gender** | **Years of Experience** | **Professional Category** |
| --- | --- | --- | --- | --- |
| Initial Expert Review | 36-45 | Female | 5-10 years | Public Health Research |
|  | 36-45 | Male | 11-20 years | TB Clinical Expert |
|  | >45 | Male | >20 years | TB Program Management |
| Healthcare Provider | 25-35 | Female | 5-10 years | Community Health Worker (n=1), DOT Provider (n=1) |
|  | 25-35 | Female | 11-20 years | Community Health Worker (n=1) |
|  | >45 | Female | 11-20 years | Community Health Worker (n=1) |
|  | 25-35 | Male | 11-20 years | Medical Officer (n=1) |
|  | >45 | Male | 11-20 years | Medical Officer (n=1), Laboratory Supervisor (n=1) |
|  | 36-45 | Male | 11-20 years | TB Program Supervisor (n=1) |
| Expert Panel Review | 36-45 | Female | 11-20 years | TB Consultant (n=1), Physician (n=1) |
|  | 36-45 | Female | >20 years | Public Health Specialist (n=1) |
|  | 36-45 | Male | 11-20 years | Development Partner (n=2), Academic Expert (n=1) |
|  | >45 | Male | 11-20 years | Development Partner (n=1), Public Health Research (n=1) |
