# Supplementary material for: Cultural adaptation and psychometric validation of the Health Literacy Instrument for Adults with Tuberculosis (HELIA-TB) in India
Source: PLoS One. 2026 Jun 30;21(6):e0352661. doi: 10.1371/journal.pone.0352661 (PMC13318012; doi:10.1371/journal.pone.0352661)
Supplement: S2 Table — (DOCX) [file pone.0352661.s002.docx]

S2 Table. Health Literacy Instrument for Adults (HELIA) – TB (Adapted Version for Research Use Only)

| **Reading** | **Never** | **Rarely** | **Sometimes** | **Usually** | **Always** |
| --- | --- | --- | --- | --- | --- |
| 1. Reading educational materials about TB (booklet, pamphlets, posters) is easy for me. | 1 | 2 | 3 | 4 | 5 |
| 2. Reading written instructions from doctor and health workers about TB is easy for me. | 1 | 2 | 3 | 4 | 5 |
| 3. Reading medical forms (such as admissions, consent, filing, etc. in hospitals and medical centers) is easy  for me. | 1 | 2 | 3 | 4 | 5 |
| 4. Reading pamphlets and instructions for  laboratory testing, sputum testing, ultrasound or radiology is easy for me. | 1 | 2 | 3 | 4 | 5 |
| **Access to information** | **Never** | **Rarely** | **Sometimes** | **Usually** | **Always** |
| 5. I can find health information from  different sources when I need such information. | 1 | 2 | 3 | 4 | 5 |
| 6. I can find health information about tuberculosis. | 1 | 2 | 3 | 4 | 5 |
| 7. I can find health information on mental  health such as depression and stress. | 1 | 2 | 3 | 4 | 5 |
| 8. I can find health information about a  specific disease when I need to. | 1 | 2 | 3 | 4 | 5 |
| 9. I can find health information for some health problems and diseases such as high blood pressure, diabetes and high  lipid levels. | 1 | 2 | 3 | 4 | 5 |
| 10. I can find health information about  harmful effects of tobacco and smoking. | 1 | 2 | 3 | 4 | 5 |
| **Understanding** | **Never** | **Rarely** | **Sometimes** | **Usually** | **Always** |
| 11. I can understand the recommendations  for a healthy diet. | 1 | 2 | 3 | 4 | 5 |
| 12. I can understand when my doctor  explains about my illness (TB). | 1 | 2 | 3 | 4 | 5 |
| 13. I can understand the meaning when reading medical forms (such as admissions, consents, filings, etc.) in  hospitals and health centers. | 1 | 2 | 3 | 4 | 5 |
| 14. I can understand signs or boards in  hospitals, clinics and health centers. | 1 | 2 | 3 | 4 | 5 |
| 15. I can understand drug information on labels | 1 | 2 | 3 | 4 | 5 |

| 16. I can understand the risks and benefits  of drugs prescribed by my doctor. | 1 | 2 | 3 | 4 | 5 |
| --- | --- | --- | --- | --- | --- |
| 17. I can understand written information  before sputum testing, ultrasound or radiology. | 1 | 2 | 3 | 4 | 5 |
| **Appraisal** | **Never** | **Rarely** | **Sometimes** | **Usually** | **Always** |
| 18. I can evaluate health-related  information on the Internet. | 1 | 2 | 3 | 4 | 5 |
| 19. I can evaluate health-related  information broadcast on television and radio. | 1 | 2 | 3 | 4 | 5 |
| 20. I can assess the accuracy of health- related recommendations I receive from  relatives and friends. | 1 | 2 | 3 | 4 | 5 |
| 21. I can communicate trusted health  information to others. | 1 | 2 | 3 | 4 | 5 |
| **Decision-making/ behavioural intention** | **Never** | **Rarely** | **Sometimes** | **Usually** | **Always** |
| 22. When facing an illness, I know where  to go or with whom to speak. | 1 | 2 | 3 | 4 | 5 |
| 23. When doctor suggests that I should take medicines three times a day, I know that I should take one tablet every  8 hours. | 1 | 2 | 3 | 4 | 5 |
| 24. I do not cut my medications without  my doctor’s permission, even if symptoms disappear. | 1 | 2 | 3 | 4 | 5 |
| 25. If anyone in my family develops cough or weight loss, I see a doctor to examine them. | 1 | 2 | 3 | 4 | 5 |
| 26. I avoid doing or eating things that  increase my blood pressure. | 1 | 2 | 3 | 4 | 5 |
| 27. I visit my doctor for regular  checkups. | 1 | 2 | 3 | 4 | 5 |
| 28. I am health-conscious in any situation. | 1 | 2 | 3 | 4 | 5 |
| 29. If needed, I ask my doctor or health  care staff questions about my disease. | 1 | 2 | 3 | 4 | 5 |
| 30. I buy food products (like oil or milk) after checking if they are healthy for someone with TB. | 1 | 2 | 3 | 4 | 5 |
| 31. I avoid substances that increases my blood pressure or sugar levels. | 1 | 2 | 3 | 4 | 5 |
| 32. I use a helmet or seat belt while driving. | 1 | 2 | 3 | 4 | 5 |
| 33. I consider the food labels when  shopping. | 1 | 2 | 3 | 4 | 5 |

Adapted from the Health Literacy Instrument for Adults (HELIA)
Original instrument: Montazeri et al., 2017

Culturally adapted and validated for adults with tuberculosis in India by:
Chauhan et al., 2026
