# Supplementary material for: Cultural adaptation and psychometric validation of the Health Literacy Instrument for Adults with Tuberculosis (HELIA-TB) in India
Source: PLoS One. 2026 Jun 30;21(6):e0352661. doi: 10.1371/journal.pone.0352661 (PMC13318012; doi:10.1371/journal.pone.0352661)
Supplement: Attachment — Submitted filename: Response to reviewers.pdf [file pone.0352661.s003.pdf]

Journal requirements	
1. Please ensure that your manuscript meets PLOS ONE's style requirements,<br>including those for file naming. The PLOS ONE style templates can be found at<br>https://journals.plos.org/plosone/s/file?id=wjVg/PLOSOne_formatting_sample_<br>main_body.pdf and<br>https://journals.plos.org/plosone/s/file?id=ba62/PLOSOne_formatting_sample_title_<br>authors_affiliations.pdf	Thank you. We have revised the manuscript as per the journal requirements.
2. Your ethics statement should only appear in the Methods section of your manuscript. If your ethics statement is written in any section besides the Methods, please move it to the Methods section and delete it from any other section. Please ensure that your ethics statement is included in your manuscript, as the ethics statement entered into the online submission form will not be published alongside your manuscript.	Addressed. Move to method section<br>The suggested changes can be found on page 18, line 373-379.
3. 3. Please ensure that you refer to Figure 1 in your text as, if accepted, production will need this reference to link the reader to the figure.	Addressed. The suggested change can be found on page 214, line 11.
4. 4. Please include captions for your Supporting Information files at the end of your manuscript, and update any in-text citations to match accordingly. Please see our Supporting Information guidelines for more information: http://journals.plos.org/plosone/s/supporting-information. – we have removed supplementary files and have included only final validated tool.	Addressed and included at the end of the manuscript. The suggested change can be found on page 46, line 785-787.
5. We note that there is identifying data in the Supporting Information file < Supplementary Table 2.docx >. Due to the inclusion of these potentially identifying data, we have removed this file from your file inventory. Prior to sharing human research participant data, authors should consult with an ethics committee to ensure data are shared in accordance with participant consent and all applicable local laws. Data sharing should never compromise participant privacy. It is therefore not appropriate to publicly share personally identifiable data on human research participants. The following are examples of data that should not be shared:<br>-Name, initials, physical address<br>-Ages more specific than whole numbers<br>-Internet protocol (IP) address<br>-Specific dates (birth dates, death dates, examination dates, etc.)<br>-Contact information such as phone number or email address<br>-Location data	We have addressed this. Thank you

-ID numbers that seem specific (long numbers, include initials, titled "Hospital ID") rather than random (small numbers in numerical order)<br>Data that are not directly identifying may also be inappropriate to share, as in combination they can become identifying. For example, data collected from a small group of participants, vulnerable populations, or private groups should not be shared if they involve indirect identifiers (such as sex, ethnicity, location, etc.) that may risk the identification of study participants. Additional guidance on preparing raw data for publication can be found in our Data Policy (https://journals.plos.org/plosone/s/data-availability#loc-human-research-participant-data-and-other-sensitive-data) and in the following article: http://www.bmj.com/content/340/bmj.c181.long.<br><br>Please remove or anonymize all personal information (ID numbers that seem specific and Ages more specific than whole numbers), ensure that the data shared are in accordance with participant consent, and re-upload a fully anonymized data set. Please note that spreadsheet columns with personal information must be removed and not hidden as all hidden columns will appear in the published file.	
6. If the reviewer comments include a recommendation to cite specific previously published works, please review and evaluate these publications to determine whether they are relevant and should be cited. There is no requirement to cite these works unless the editor has indicated otherwise	Thank you for this clarification. We confirm that no reviewer specifically recommended inclusion of additional citations or previously published works. Accordingly, no changes were made in this regard.
Reviewer Comments	
1- "Study Participants": Describe participants (mean age (SD), working history, etc), if possible.	Thank you for this valuable suggestion. We have revised the "Study Participants" section to provide a clearer description of participant characteristics across all stages of adaptation and validation. Specifically, we have now included details on participants' age ranges, years of professional experience for experts and healthcare providers, as well as demographic and clinical characteristics of TB patient participants, including mean age (SD), gender distribution, education status, comorbidity profile, and healthcare setting. In addition, we clarified that detailed characteristics of experts and healthcare providers are presented in S1 Table.<br>The suggested changes can be found on page 9-10, line 171-194.
2- It is better to add an explanation about the ratio of the sample size (393 participants). For example, point out the number of questions in HELIA.	Thank you for this important suggestion. We have now clarified the rationale for the validation sample size in the "Sample size and sampling" subsection. Specifically, we added that the HELIA-TB instrument contains 33 items, and the validation sample of 393 participants exceeded the commonly recommended ratio of 5–10 participants per item for psychometric validation and factor-analytic procedures. This

	strengthened the statistical power and adequacy of the validation analyses.<br>The suggested changes can be found on page 145, line 286-299
3- SPSS version 26 was used for quantitative analysis. Have you done CFA in SPSS?<br>If yes, explain in the manuscript or add the SPSS scripts to the supplementary materials.<br>(Usually, CFA HAVE DONE WITH AMOS, LISREL, or Smart-PLS).	Thank you for this observation. Confirmatory Factor Analysis (CFA) was conducted using the AMOS module available within the SPSS version 26 environment. We have now clarified this in the “Data Analysis” subsection of the manuscript by specifying the software and estimation method used for CFA.<br>The suggested change can be found on page 16, line 338-340.
4- If it's possible, rewrite and redesign Tables 1 and 2 to be small.	Thank you for this suggestion. To improve the readability and conciseness of the manuscript, we have moved Table 1 (detailed characteristics of experts and healthcare providers) to the supplementary materials as Supplementary S1 Table. Table 2 was retained in the main manuscript because the item-level content validity assessment is central to the psychometric validation process.<br>The suggested change can be found in S1 Table.
5- For the RMSEA index, report 95% Confidence Interval.	Thank you for this valuable suggestion. We agree that reporting the confidence interval for RMSEA enhances the interpretability and completeness of CFA reporting. Accordingly, the manuscript has been revised to include the RMSEA confidence interval.<br><br>The suggested change can be found on Page 34, line 540.
6- Check Link for Ref 14. I cannot access the link.	Thank you for highlighting this issue. We rechecked the link/reference for Ref. 14 and confirmed that it is accessible and functioning correctly from our side. Nevertheless, we have carefully verified the URL and reference formatting in the revised manuscript to ensure accessibility.
Reviewer #2	
: 1. Title and Abstract<br>The title is structurally appropriate; however, the phrase “Cultural Adaptation and Validation” could be made more precise by indicating the type of validation conducted (e.g., confirmatory factor analysis, predictive validity), particularly since the manuscript reports CFA and multiple forms of construct validity. These important methodological components are not reflected in the title.	Thank you for this valuable suggestion. We agree that the title should better reflect the broader psychometric evaluation conducted in the study, including confirmatory factor analysis and construct validity assessments. Accordingly, we have revised the title to:<br>“Cultural Adaptation and Psychometric Validation of the Health Literacy Instrument for Adults with Tuberculosis (HELIA-TB) in India.”

In the abstract, the study is described as an “exploratory sequential mixed-methods design,” yet the operational linkage between the qualitative and quantitative phases is not clearly explained. It remains unclear how qualitative findings directly informed the quantitative validation or how integration occurred. Furthermore, the timeframe (March 2024–April 2025) raises concerns, as it overlaps with the manuscript submission period; clarification is required regarding whether data collection was fully completed prior to submission.	Thank you for this thoughtful observation. We have revised the abstract and Methods section to clarify the operational linkage between the qualitative and quantitative phases of the exploratory sequential mixed-methods design. Specifically, we now explain that findings from the qualitative phase, including expert review and cognitive interviews with TB patients and healthcare providers, directly informed item refinement, contextual adaptation, terminology simplification, and cultural modifications prior to quantitative psychometric validation. The revised instrument was subsequently evaluated through reliability and construct validity analyses in the larger quantitative sample.<br>The suggested change can be found on page 3, 52-60.<br><br>We also clarified the study timeline. Participant recruitment and data collection were completed prior to manuscript submission. The reported study period (March 2024–April 2025) reflects the overall duration of the study, including data cleaning, analysis, interpretation, and manuscript preparation following submission. We have now revised this to March 2024-March<br>The suggested change can be found on page 3, line 53-54.
The abstract reports effect sizes (Cohen’s d), but the scoring range and scaling method of the HELIA-TB are not specified, limiting interpretability of the magnitude of these effects. Additionally, the statement that no significant differences were observed by TB type or drug resistance status does not acknowledge the extremely small sample size in the DR-TB group (n=11), which substantially increases the risk of Type II error.	Thank you for these important observations. Additional clarification regarding HELIA-TB scoring has now been incorporated into the revised manuscript. Consistent with the original HELIA scoring protocol, raw scores were linearly transformed to a standardized 0–100 scale, with higher scores indicating higher health literacy levels, thereby improving interpretability of reported effect sizes.<br>The suggested change can be found on page 16-16, line 336-338.<br><br>We also acknowledge that the relatively small number of participants with drug-resistant TB (n = 11) substantially limited statistical power for subgroup comparisons and increased the possibility of Type II error. Accordingly, findings related to drug resistance status have been interpreted cautiously and this limitation has now been acknowledged in the manuscript.<br>The suggested change can be found on page 39-40, line 644-657.

The conclusion in the abstract states that the tool "has potential to enhance adherence," which implies a causal relationship that cannot be supported by the cross-sectional design. This wording should be tempered.	Thank you for this important observation. The conclusion in the abstract has been revised to avoid implying a causal relationship between the instrument and treatment adherence. The wording has been tempered to emphasize the tool's potential role in health literacy assessment and in informing targeted TB care interventions rather than directly enhancing adherence outcomes.<br>The suggested change can be found on page 4, line 76-78.
2. Introduction<br><br>The epidemiological statistics on TB incidence and mortality in India are presented without specifying the reference year, which is critical given annual variations in TB reporting.	Thank you for highlighting this. We have revised and now included the reference year. The suggested change can be found on page 5, line 85.
The assertion of a "growing consensus" favoring adaptation over new tool development is not adequately substantiated with comparative methodological or empirical evidence.	Thank you for this important observation. The wording has been revised to avoid overstating the level of consensus regarding adaptation approaches. The revised text now emphasizes that cultural adaptation and validation of established instruments is increasingly recognized as a practical and scientifically efficient strategy because it allows contextual relevance while maintaining conceptual comparability across settings and populations.<br><br>The suggested change can be found on page 6-7, line 126-129.
The manuscript states that HELIA was "developed in Iran in English," which appears factually inaccurate. HELIA was originally developed in Persian and later translated into English. This point is important because it directly affects the justification of the translation and adaptation pathway.	Response: Thank you for this important observation. The manuscript has been clarified to better describe the adaptation pathway used in the present study. HELIA was originally developed in Iran, and the English version provided by the original developers was used as the reference version during the cultural adaptation process for the present study. This clarification has been incorporated to improve transparency regarding translation and adaptation procedures.<br>The suggested change can be found on Page 8, line 156-158.
The comparison with instruments such as HLQ and HLS-EU emphasizes licensing constraints, but the theoretical and structural differences between these tools and HELIA are insufficiently analyzed. The rationale for selecting HELIA appears more pragmatic (open access) than theoretically grounded.	Thank you for this important observation. The selection of HELIA was based not solely on considerations of accessibility, but on its conceptual structure, multidimensional framework, and contextual relevance for TB care within a lower-middle-income country setting. Unlike instruments such as REALM and TOFHLA, which primarily assess functional literacy skills, HELIA evaluates broader health literacy competencies including access to information, reading, understanding, appraisal,

	and decision-making, thereby aligning more closely with contemporary multidimensional health literacy frameworks and the practical self-management demands associated with TB treatment. Although HLQ and HLS-EU are comprehensive instruments with strong psychometric foundations, they were developed within substantially different sociocultural and health-system contexts and involve adaptation procedures that may include licensing restrictions, administrative permissions, and collaborative requirements that can limit independent contextual adaptation in resource-constrained settings. In contrast, HELIA was originally developed and validated in a lower-middle-income country context with healthcare and literacy realities more comparable to the present study population. Therefore, the rationale for selecting HELIA was both theoretically grounded and contextually informed, rather than based solely on pragmatic considerations.
3. Methods<br>Study Design<br><br>Although the study is described as an exploratory sequential mixed-methods design, the integration strategy is not adequately articulated. The manuscript does not explain how qualitative findings shaped item refinement in a traceable, systematic manner. No joint display or explicit integration framework is presented.	Thank you for this important observation. The exploratory sequential mixed-methods integration strategy was operationalized through iterative use of qualitative findings to guide refinement of the HELIA-TB instrument prior to quantitative psychometric validation. As described in the manuscript, findings from expert review and cognitive interviews directly informed item modification, terminology simplification, contextual adaptation, and refinement of culturally sensitive wording. In addition, the Results section provides concrete examples of feedback-driven revisions and corresponding item adaptations, thereby demonstrating the integration of qualitative insights into development of the final quantitative instrument. Given the primarily instrument-adaptation focus of the study, a formal joint display framework was not developed.
Sampling<br><br>The claim of simple random sampling from TB registers lacks detail regarding the sampling frame, total eligible population, response rate, and potential selection bias. Without this information, representativeness cannot be assessed.	Thank you for this important observation. Additional clarification regarding the sampling procedure has now been incorporated into the revised manuscript.<br><br>The suggested change can be found on page 15, line 304-312.
The justification of sample size using the rule of 5–10 participants per item is more appropriate for exploratory factor analysis than for CFA using maximum likelihood estimation. A more rigorous justification specific to CFA is required.	Thank you for this important observation. We agree that the 5–10 participants per item rule is more commonly applied to exploratory factor analysis and provides only a general reference for psychometric studies. The manuscript has therefore been revised to provide a CFA-specific

	justification based on model complexity, maximum likelihood estimation requirements, and recommended minimum sample thresholds for structural equation modeling. The final validation sample of 393 participants was considered adequate for the 33-item, five-factor CFA model and exceeded commonly recommended minimum sample sizes for stable CFA estimation.<br>The suggested change can be found on page 14, line 285-299.
The DR-TB subgroup (n=11) is too small to support meaningful discriminant validity testing. Conducting t-tests with such a small comparison group raises serious concerns about statistical power, yet this limitation is not adequately discussed.	Thank you for this important observation. We acknowledge that the number of participants with drug-resistant TB was relatively small (n = 11), which limits the statistical power and precision of subgroup comparisons involving drug resistance status. The discriminant validity analysis related to DR-TB was intended as an exploratory assessment rather than a definitive comparison. This limitation and the need for further validation in larger DR-TB samples have now been acknowledged in the revised manuscript.<br>The suggested change can be found on page 39-40, line 644-657.
Cultural Adaptation Process<br><br>Only five patients participated in cognitive interviews, which is insufficient to robustly claim data saturation. The argument that subsequent large-scale validation compensates for limited cognitive testing is methodologically flawed, as the purpose of cognitive interviewing is to identify conceptual and interpretative issues before quantitative testing.	Thank you for this important methodological observation. Although cognitive interviews were conducted with five TB patients, the adaptation process additionally incorporated input from eight healthcare providers involved in TB care delivery, thereby allowing assessment of both patient comprehension and practical field applicability of the instrument. Participants were purposively selected to capture variability in literacy level, treatment experience, and contextual understanding relevant to the target population. Given the intensive nature of cognitive interviewing and the clinical vulnerability of patients undergoing TB treatment, this focused qualitative approach was considered appropriate for preliminary cultural adaptation and conceptual evaluation of the HELIA-TB instrument.
Although HSRI guidelines are referenced, the specific steps followed and how they were operationalized are not clearly described.	Thank you for this observation. The manuscript already outlines the operationalization of the HSRI-guided adaptation process across multiple sequential phases, including expert review, forward translation, cognitive interviewing, expert panel review, back translation, pilot testing, and psychometric validation. In addition to describing these stages, the manuscript also details how expert and participant feedback informed item-level revisions, terminology simplification, contextual adaptation, and

	refinement of culturally sensitive wording for the TB care context. Concrete examples of modified items and translation adjustments were also provided to demonstrate the practical implementation of the adaptation framework.
It is also unclear whether think-aloud techniques or structured probing were systematically used during cognitive interviews.	Thank you for this important methodological observation. During the cognitive interviewing phase, semi-structured probing techniques were systematically used to explore participant comprehension, interpretation, clarity, and emotional acceptability of individual items. Participants were encouraged to explain questions in their own words, identify difficult terminology, and describe how they interpreted key concepts within the TB care context. Although formal think-aloud procedures were not employed in a highly structured manner, iterative probing and follow-up questioning were used consistently throughout the interviews to guide item refinement and contextual adaptation.
Content Validity<br><br>While I-CVI and S-CVI/Ave are reported, modified kappa coefficients were not calculated to adjust for chance agreement. The proportion of experts rating each item as 3 or 4 (for relevance) is not explicitly reported.	Thank you for this valuable methodological observation. In the revised manuscript, additional clarification has been provided regarding the content validity procedure. The Item-Level Content Validity Index (I-CVI) was calculated as the proportion of experts rating each item as either “quite relevant” or “highly relevant” on the relevance scale, with observed I-CVI values ranging from 0.88 to 1.00. While modified kappa coefficients to adjust for chance agreement were not calculated in the present study, the consistently high I-CVI and S-CVI/Ave values indicated strong expert agreement regarding item relevance and clarity. This limitation has now been acknowledged in the manuscript, and future studies may incorporate modified kappa statistics for more rigorous content validity assessment.<br><br>The suggested change can be found on page 39-40, line 644-657.
Reliability<br><br>Cronbach’s alpha is reported for the overall scale despite the multidimensional structure. The appropriateness of reporting an overall alpha for a five-factor model requires theoretical justification.	Thank you for this important observation. While the HELIA-TB instrument comprises five theoretically distinct domains, the overall Cronbach’s alpha was reported to reflect the internal consistency of the instrument when used as a comprehensive multidimensional health literacy measure. In addition, domain-specific Cronbach’s alpha values were also calculated and presented separately in Table 3 to account for the multidimensional structure of the scale.

The 30-day interval for test–retest reliability may allow for real changes in health literacy during TB treatment, particularly as patients receive ongoing counseling. This potential source of variability is not discussed.	Thank you for this important observation. We acknowledge that the 30-day interval used for test–retest reliability assessment may have permitted genuine changes in health literacy levels, particularly because participants were undergoing active TB treatment and receiving ongoing counseling and health education during this period. This potential source of variability has now been acknowledged in the limitations section of the revised manuscript.<br><br>The suggested change can be found on page 39-40, line 644-657.
Confirmatory Factor Analysis<br><br>CFA was conducted using maximum likelihood estimation, but no evidence is provided regarding multivariate normality assumptions. The chi-square statistic and χ^2/df ratio are not reported. There is no discussion of modification indices or correlated residuals.<br><br>One item (Q32) demonstrated a low factor loading ($\lambda = 0.43$), yet was retained without robust statistical justification. The decision appears primarily theoretical and requires stronger defense.	Thank you for these important methodological observations. Additional clarification has now been incorporated into the revised manuscript. CFA was conducted using maximum likelihood estimation after preliminary assessment of the data distribution, including inspection of skewness, kurtosis, and inter-item correlations, which supported the suitability of the estimation approach for the present sample size. The overall model fit statistics have now been expanded to include the chi-square statistic and χ^2/df ratio alongside CFI, TLI, RMSEA, and SRMR.<br>The final CFA model was refined iteratively based on theoretical interpretability and model diagnostics, including examination of modification indices and correlated residuals between conceptually related items. These refinements were undertaken cautiously to preserve the theoretical structure of the original HELIA framework while improving model fit within the Indian TB care context.<br><br>Regarding Q32, although the standardized factor loading was relatively lower ($\lambda = 0.43$), the item was retained due to its conceptual relevance to preventive health decision-making behaviors and because its inclusion did not substantially reduce the overall composite reliability or internal consistency of the scale. Retaining theoretically meaningful items despite marginal loadings is consistent with recommendations in psychometric validation literature, particularly in culturally adapted instruments.<br><br>The suggested changes can be found on page 34, line 538-553.

Although composite reliability (CR) and AVE were reported, discriminant validity between latent constructs was not assessed using Fornell–Larcker criteria or HTMT ratios.	Thank you for this important observation. In the revised manuscript, additional clarification has been added regarding construct validity assessment. While formal Fornell–Larcker and HTMT analyses were not originally performed, discriminant validity was evaluated through examination of inter-factor correlations alongside AVE estimates derived from the CFA model. The observed correlations between latent constructs remained theoretically coherent and below levels typically suggestive of construct redundancy, supporting acceptable conceptual distinction between domains. This limitation and the need for further advanced discriminant validity assessment in future studies have now been acknowledged in the manuscript.<br><br>The suggested change can be found on page 39-40, line 644-657.
4. Results<br><br>The item-total correlation range includes an extremely low value (0.067), suggesting weak alignment with the overall construct. Retaining such an item without conducting sensitivity analyses or reporting alternative models is problematic.	Thank you for this important observation. We acknowledge that Q32 demonstrated a relatively low item-total correlation and factor loading compared with other items. Sensitivity analyses examining the effect of item removal on internal consistency were reviewed and indicated only minimal improvement in overall reliability estimates. Given its conceptual relevance to preventive health and safety-related decision-making behaviors, the item was retained to preserve construct breadth and theoretical coherence of the adapted HELIA-TB instrument.
Predictive validity was assessed using independent t-tests without controlling for potential confounders such as age, education, gender, or duration of treatment. Therefore, conclusions about predictive validity remain limited.	Thank you for this important methodological observation. We acknowledge that predictive validity analyses were conducted using unadjusted group comparisons and did not account for potential confounding factors such as age, gender, educational status, or duration of treatment. The predictive validity findings have therefore been interpreted cautiously as preliminary evidence, and this limitation has now been acknowledged in the revised manuscript.<br>The suggested change can be found on page 39-40, line 644-657.
Convergent validity relied on a single-item self-rated health measure, which has inherent measurement limitations. This methodological constraint is not sufficiently acknowledged.	Thank you for this observation. We acknowledge that convergent validity was assessed using a single-item self-rated health measure, which has inherent measurement limitations compared with multidimensional health assessment tools. This limitation has now been acknowledged in the revised

	manuscript and the findings have been interpreted accordingly.<br>The suggested change can be found on page 39-40, line 644-657.
There are minor inconsistencies in reported degrees of freedom in some t-tests relative to group sample sizes, suggesting the need for statistical verification.	Thank you for identifying this issue. The reported degrees of freedom in the t-test results have been reviewed and corrected where necessary to ensure consistency with the corresponding group sample sizes and statistical procedures used in the analyses.<br>The suggested changes can be found on Page 31, line 499, page 32, line 507 and 513.
5. Discussion<br><br>The discussion occasionally implies causal interpretation of associations between health literacy and adherence, despite the cross-sectional design.	Thank you for this important observation. The Discussion section has been revised to avoid causal interpretation of associations identified in the study.<br>The suggested changes can be found on page 36-38.
The comparison of the observed effect size (d=0.89) with meta-analytic estimates from other chronic diseases does not sufficiently account for differences in instruments, adherence measurement methods, and disease contexts. This comparison may overstate the strength of the findings.	Thank you for this observation. The comparison with previously reported meta-analytic effect sizes was intended to provide contextual interpretation of the observed association rather than to imply direct equivalence across disease conditions or measurement approaches. The manuscript already acknowledges the complexity and distinct treatment burden associated with TB, which may influence the strength of health literacy–adherence relationships. Nevertheless, we have revised the wording to further emphasize that differences in instruments, adherence measures, and clinical contexts should be considered when interpreting cross-study comparisons.<br>The suggested change can be found on page 38, line 617-619.
The claim of superiority over other TB literacy tools based solely on S-CVI comparisons is not methodologically sound, as content validity indices are not directly comparable across studies with different expert panels and contexts.<br>Thank you for this important methodological observation. We agree that direct comparison of content validity indices across studies should be interpreted cautiously because expert panel composition, contextual factors, and adaptation procedures may differ substantially. The manuscript has therefore been revised to avoid implying methodological superiority based solely on S-CVI comparisons and now presents these findings as broadly comparable contextual reference points.	Thank you for this important methodological observation. We agree that direct comparison of content validity indices across studies should be interpreted cautiously because expert panel composition, contextual factors, and adaptation procedures may differ substantially. The manuscript has therefore been revised to avoid implying methodological superiority based solely on S-CVI comparisons and now presents these findings as broadly comparable contextual reference points.<br>The suggested change can be found on page 39, line 609.
Limitations are acknowledged but not deeply analyzed. In particular, the following require stronger emphasis:<br><br>Single-district validation,<br><br>Potential social desirability bias,	Thank you for these important observations. The limitations section has been revised and strengthened to provide a more balanced discussion of methodological constraints. Specifically, the revised manuscript now more clearly acknowledges the single-district validation setting, the relatively small DR-TB

Lack of measurement invariance testing,<br><br>Absence of responsiveness analysis,<br><br>Small DR-TB subgroup size.<br><br>6. Structural and Reporting Issues	subgroup size and its implications for statistical power, the absence of formal measurement invariance testing and responsiveness analysis, the potential influence of social desirability bias associated with self-reported responses, and limitations related to cross-sectional design and unadjusted predictive validity analyses. Additional clarification regarding the 30-day test–retest interval, convergent validity assessment, and generalizability has also been incorporated. These revisions were made to improve methodological transparency and provide clearer direction for future validation research.<br>The suggested change can be found on page 39-40, line 644-657.
There are minor inconsistencies in statistical reporting (e.g., degrees of freedom) that require careful verification.	Thank you. We have now addressed this and revise the inconsistencies in statistical reporting across the manuscript.
Frequent use of evaluative descriptors such as “excellent,” “robust,” and “strong” is not always anchored in clearly defined benchmarks.	Thank you for this observation. The evaluative descriptors used throughout the manuscript (e.g., “excellent,” “strong,” and “robust”) were intended to reflect commonly accepted psychometric interpretation benchmarks for indices such as Cronbach’s alpha, ICC, CFA fit statistics, and effect sizes. Nevertheless, to improve precision and avoid overstatement, several descriptors have been moderated or linked more explicitly to the corresponding quantitative findings and established interpretive criteria in the revised manuscript.
Some adaptation decisions (e.g., replacing TB-specific wording with broader “health condition” terminology) may weaken the TB-specific focus of the instrument and require clearer theoretical justification.	The modification of selected wording from “TB” to the broader term “health condition” was intentional and informed by findings from cognitive interviews. Participants and frontline healthcare workers indicated that repeated direct references to TB could evoke discomfort, stigma, or reluctance during discussion of sensitive health behaviors. Broader terminology was therefore used selectively in items where the underlying construct related to general health decision-making or healthcare navigation rather than TB-specific clinical knowledge. This approach helped preserve conceptual equivalence with the original HELIA domains, improve participant comfort and acceptability, and maintain applicability among patients with TB and coexisting chronic conditions.
