# Supplementary material for: Cultural adaptation and psychometric validation of the Health Literacy Instrument for Adults with Tuberculosis (HELIA-TB) in India
Source: PLoS One. 2026 Jun 30;21(6):e0352661. doi: 10.1371/journal.pone.0352661 (PMC13318012; doi:10.1371/journal.pone.0352661)
Supplement: Attachment — Submitted filename: Response to Editor.docx [file pone.0352661.s004.docx]

Dear Editor in Chief

After reviewing the authors’ responses, I would like to provide the following comments:

1- "Study Participants": Describe participants (mean age (SD), working history, etc), if possible.

This issue has been addressed. However, it would be preferable to present the information in a clearer table format. At minimum, reduce row lines to improve table readability.

2- It is better to add an explanation about the ratio of the sample size (393 participants).

This issue has been clarified.

3- SPSS version 26 was used for quantitative analysis. Have you done CFA in SPSS?

This issue has been addressed.

4- If it's possible, rewrite and redesign Tables 1 and 2 to be small.

This issue has been addressed.

5- For the RMSEA index, report 95% Confidence Interval.

This issue has been clarified.

6- Check Link for Ref 14. I cannot access the link

Reference 14 is currently presented as:
“14. Directorate of Census Operations, Gujarat”

References, especially reference 14, need to be checked again based on PloS ONE style.

I would like to thank the respected editor of the journal for inviting me to be a referee.

Good Luck.
