# Supplementary material for: Cultural adaptation and psychometric validation of the Health Literacy Instrument for Adults with Tuberculosis (HELIA-TB) in India
Source: PLoS One. 2026 Jun 30;21(6):e0352661. doi: 10.1371/journal.pone.0352661 (PMC13318012; doi:10.1371/journal.pone.0352661)
Supplement: Attachment — Submitted filename: REsponse to reviewer_R2.docx [file pone.0352661.s006.docx]

Thank you for your careful review and constructive suggestions.

We have revised the manuscript in accordance with the recommendations. Specifically:

1. **PLOS ONE citation and reference style:** All in-text citations and references were reviewed and reformatted to align with PLOS ONE guidelines. Reference formatting, author listings, journal abbreviations, punctuation, and web-based citations were checked and corrected where necessary.
2. **Use of abbreviations:** Repeated use of terms with both full forms and abbreviations has been corrected. Terms are now defined at first mention and abbreviated forms are used consistently throughout the remainder of the manuscript.
3. **Language and grammar:** The manuscript has undergone a comprehensive proofreading for grammar, punctuation, consistency, and readability. The unnecessary quotation marks identified by the reviewer have been removed, and several minor language edits have been made throughout the text.
4. **Tables and figures:** All tables and figures were reviewed for consistency, formatting, clarity, and alignment with journal requirements. Minor formatting corrections were implemented where needed.
