# Supplementary material for: Cultural adaptation and psychometric validation of the Health Literacy Instrument for Adults with Tuberculosis (HELIA-TB) in India
Source: PLoS One. 2026 Jun 30;21(6):e0352661. doi: 10.1371/journal.pone.0352661 (PMC13318012; doi:10.1371/journal.pone.0352661)
Supplement: Peer review history

# Peer review: Decision Letter 0

**DOI:** 10.1371/journal.pone.0352661.r001

**Authors:** Muhammad Haroon Stanikzai

14 Apr 2026

PONE-D-26-06515

Cultural Adaptation and Validation of the Health Literacy Instrument for Adults with Tuberculosis (HELIA-TB) in India

PLOS One

Dear Dr. Chauhan,

Thank you for submitting your manuscript to PLOS ONE. After careful consideration, we feel that it has merit but does not fully meet PLOS ONE’s publication criteria as it currently stands. Therefore, we invite you to submit a revised version of the manuscript that addresses the points raised during the review process.

==============================

Two review reports have been obtained. Please revised the manuscript based on estemeed reviewers' suggestions and comments.

==============================

Please submit your revised manuscript by May 29 2026 11:59PM. If you will need more time than this to complete your revisions, please reply to this message or contact the journal office at plosone@plos.org. When you're ready to submit your revision, log on to https://www.editorialmanager.com/pone/ and select the 'Submissions Needing Revision' folder to locate your manuscript file.

Please include the following items when submitting your revised manuscript:

- A letter that responds to each point raised by the academic editor and reviewer(s). You should upload this letter as a separate file labeled 'Response to Reviewers'.
- A marked-up copy of your manuscript that highlights changes made to the original version. You should upload this as a separate file labeled 'Revised Manuscript with Track Changes'.
- An unmarked version of your revised paper without tracked changes. You should upload this as a separate file labeled 'Manuscript'.

If you would like to make changes to your financial disclosure, please include your updated statement in your cover letter. Guidelines for resubmitting your figure files are available below the reviewer comments at the end of this letter.

If applicable, we recommend that you deposit your laboratory protocols in protocols.io to enhance the reproducibility of your results. Protocols.io assigns your protocol its own identifier (DOI) so that it can be cited independently in the future. For instructions see: https://journals.plos.org/plosone/s/submission-guidelines#loc-laboratory-protocols. Additionally, PLOS ONE offers an option for publishing peer-reviewed Lab Protocol articles, which describe protocols hosted on protocols.io. Read more information on sharing protocols at https://plos.org/protocols?utm_medium=editorial-email&utm_source=authorletters&utm_campaign=protocols.

As the corresponding author, your ORCID iD is verified in the submission system and will appear in the published article. PLOS supports the use of ORCID, and we encourage all coauthors to register for an ORCID iD and use it as well. Please encourage your coauthors to verify their ORCID iD within the submission system before final acceptance, as unverified ORCID iDs will not appear in the published article. *Only*  the individual author can complete the verification step; PLOS staff *cannot*  verify ORCID iDs on behalf of authors.

We look forward to receiving your revised manuscript.

Kind regards,

Muhammad Haroon Stanikzai

Academic Editor

PLOS One

**Journal Requirements:**

When submitting your revision, we need you to address these additional requirements.

1. Please ensure that your manuscript meets PLOS ONE's style requirements, including those for file naming. The PLOS ONE style templates can be found at

https://journals.plos.org/plosone/s/file?id=wjVg/PLOSOne_formatting_sample_main_body.pdf and

https://journals.plos.org/plosone/s/file?id=ba62/PLOSOne_formatting_sample_title_authors_affiliations.pdf

2. Your ethics statement should only appear in the Methods section of your manuscript. If your ethics statement is written in any section besides the Methods, please move it to the Methods section and delete it from any other section. Please ensure that your ethics statement is included in your manuscript, as the ethics statement entered into the online submission form will not be published alongside your manuscript.

3. Please ensure that you refer to Figure 1 in your text as, if accepted, production will need this reference to link the reader to the figure.

4. Please include captions for your Supporting Information files at the end of your manuscript, and update any in-text citations to match accordingly. Please see our Supporting Information guidelines for more information: http://journals.plos.org/plosone/s/supporting-information.

5. We note that there is identifying data in the Supporting Information file < Supplementary Table 2.docx >. Due to the inclusion of these potentially identifying data, we have removed this file from your file inventory. Prior to sharing human research participant data, authors should consult with an ethics committee to ensure data are shared in accordance with participant consent and all applicable local laws.

Data sharing should never compromise participant privacy. It is therefore not appropriate to publicly share personally identifiable data on human research participants. The following are examples of data that should not be shared:

-Name, initials, physical address

-Ages more specific than whole numbers

-Internet protocol (IP) address

-Specific dates (birth dates, death dates, examination dates, etc.)

-Contact information such as phone number or email address

-Location data

-ID numbers that seem specific (long numbers, include initials, titled “Hospital ID”) rather than random (small numbers in numerical order)

Data that are not directly identifying may also be inappropriate to share, as in combination they can become identifying. For example, data collected from a small group of participants, vulnerable populations, or private groups should not be shared if they involve indirect identifiers (such as sex, ethnicity, location, etc.) that may risk the identification of study participants.

Additional guidance on preparing raw data for publication can be found in our Data Policy (https://journals.plos.org/plosone/s/data-availability#loc-human-research-participant-data-and-other-sensitive-data) and in the following article: http://www.bmj.com/content/340/bmj.c181.long.

Please remove or anonymize all personal information (ID numbers that seem specific and Ages more specific than whole numbers), ensure that the data shared are in accordance with participant consent, and re-upload a fully anonymized data set. Please note that spreadsheet columns with personal information must be removed and not hidden as all hidden columns will appear in the published file.

6. If the reviewer comments include a recommendation to cite specific previously published works, please review and evaluate these publications to determine whether they are relevant and should be cited. There is no requirement to cite these works unless the editor has indicated otherwise.

[Note: HTML markup is below. Please do not edit.]

Reviewers' comments:

Reviewer's Responses to Questions

**Comments to the Author**

1. Is the manuscript technically sound, and do the data support the conclusions?

The manuscript must describe a technically sound piece of scientific research with data that supports the conclusions. Experiments must have been conducted rigorously, with appropriate controls, replication, and sample sizes. The conclusions must be drawn appropriately based on the data presented.

Reviewer #1: Yes

Reviewer #2: Yes

**********

2. Has the statistical analysis been performed appropriately and rigorously?

Reviewer #1: Yes

Reviewer #2: No

**********

3. Have the authors made all data underlying the findings in their manuscript fully available?

The PLOS Data policy requires authors to make all data underlying the findings described in their manuscript fully available without restriction, with rare exception (please refer to the Data Availability Statement in the manuscript PDF file). The data should be provided as part of the manuscript or its supporting information, or deposited to a public repository. For example, in addition to summary statistics, the data points behind means, medians and variance measures should be available. If there are restrictions on publicly sharing data—e.g. participant privacy or use of data from a third party—those must be specified.

Reviewer #1: Yes

Reviewer #2: Yes

**********

4. Is the manuscript presented in an intelligible fashion and written in standard English?

PLOS ONE does not copyedit accepted manuscripts, so the language in submitted articles must be clear, correct, and unambiguous. Any typographical or grammatical errors should be corrected at revision, so please note any specific errors here.

Reviewer #1: Yes

Reviewer #2: No

**********

5. Review Comments to the Author

Please use the space provided to explain your answers to the questions above. You may also include additional comments for the author, including concerns about dual publication, research ethics, or publication ethics. (Please upload your review as an attachment if it exceeds 20,000 characters)

Reviewer #1: There are some notes:

1- "Study Participants": Describe participants (mean age (SD), working history, etc), if possible.

2- It is better to add an explanation about the ratio of the sample size (393 participants). For example, point out the number of questions in HELIA.

3- SPSS version 26 was used for quantitative analysis. Have you done CFA in SPSS?

If yes, explain in the manuscript or add the SPSS scripts to the supplementary materials.

(Usually, CFA HAVE DONE WITH AMOS, LISREL, or Smart-PLS).

4- If it's possible, rewrite and redesign Tables 1 and 2 to be small.

5- For the RMSEA index, report 95% Confidence Interval.

6- Check Link for Ref 14. I cannot access the link.

Reviewer #2: 1. Title and Abstract

The title is structurally appropriate; however, the phrase “Cultural Adaptation and Validation” could be made more precise by indicating the type of validation conducted (e.g., confirmatory factor analysis, predictive validity), particularly since the manuscript reports CFA and multiple forms of construct validity. These important methodological components are not reflected in the title.

In the abstract, the study is described as an “exploratory sequential mixed-methods design,” yet the operational linkage between the qualitative and quantitative phases is not clearly explained. It remains unclear how qualitative findings directly informed the quantitative validation or how integration occurred. Furthermore, the timeframe (March 2024–April 2025) raises concerns, as it overlaps with the manuscript submission period; clarification is required regarding whether data collection was fully completed prior to submission.

The abstract reports effect sizes (Cohen’s d), but the scoring range and scaling method of the HELIA-TB are not specified, limiting interpretability of the magnitude of these effects. Additionally, the statement that no significant differences were observed by TB type or drug resistance status does not acknowledge the extremely small sample size in the DR-TB group (n=11), which substantially increases the risk of Type II error.

The conclusion in the abstract states that the tool “has potential to enhance adherence,” which implies a causal relationship that cannot be supported by the cross-sectional design. This wording should be tempered.

2. Introduction

The epidemiological statistics on TB incidence and mortality in India are presented without specifying the reference year, which is critical given annual variations in TB reporting.

The assertion of a “growing consensus” favoring adaptation over new tool development is not adequately substantiated with comparative methodological or empirical evidence.

The manuscript states that HELIA was “developed in Iran in English,” which appears factually inaccurate. HELIA was originally developed in Persian and later translated into English. This point is important because it directly affects the justification of the translation and adaptation pathway.

The comparison with instruments such as HLQ and HLS-EU emphasizes licensing constraints, but the theoretical and structural differences between these tools and HELIA are insufficiently analyzed. The rationale for selecting HELIA appears more pragmatic (open access) than theoretically grounded.

3. Methods

Study Design

Although the study is described as an exploratory sequential mixed-methods design, the integration strategy is not adequately articulated. The manuscript does not explain how qualitative findings shaped item refinement in a traceable, systematic manner. No joint display or explicit integration framework is presented.

Sampling

The claim of simple random sampling from TB registers lacks detail regarding the sampling frame, total eligible population, response rate, and potential selection bias. Without this information, representativeness cannot be assessed.

The justification of sample size using the rule of 5–10 participants per item is more appropriate for exploratory factor analysis than for CFA using maximum likelihood estimation. A more rigorous justification specific to CFA is required.

The DR-TB subgroup (n=11) is too small to support meaningful discriminant validity testing. Conducting t-tests with such a small comparison group raises serious concerns about statistical power, yet this limitation is not adequately discussed.

Cultural Adaptation Process

Only five patients participated in cognitive interviews, which is insufficient to robustly claim data saturation. The argument that subsequent large-scale validation compensates for limited cognitive testing is methodologically flawed, as the purpose of cognitive interviewing is to identify conceptual and interpretative issues before quantitative testing.

Although HSRI guidelines are referenced, the specific steps followed and how they were operationalized are not clearly described.

It is also unclear whether think-aloud techniques or structured probing were systematically used during cognitive interviews.

Content Validity

While I-CVI and S-CVI/Ave are reported, modified kappa coefficients were not calculated to adjust for chance agreement. The proportion of experts rating each item as 3 or 4 (for relevance) is not explicitly reported.

Reliability

Cronbach’s alpha is reported for the overall scale despite the multidimensional structure. The appropriateness of reporting an overall alpha for a five-factor model requires theoretical justification.

The 30-day interval for test–retest reliability may allow for real changes in health literacy during TB treatment, particularly as patients receive ongoing counseling. This potential source of variability is not discussed.

Confirmatory Factor Analysis

CFA was conducted using maximum likelihood estimation, but no evidence is provided regarding multivariate normality assumptions. The chi-square statistic and χ²/df ratio are not reported. There is no discussion of modification indices or correlated residuals.

One item (Q32) demonstrated a low factor loading (λ = 0.43), yet was retained without robust statistical justification. The decision appears primarily theoretical and requires stronger defense.

Although composite reliability (CR) and AVE were reported, discriminant validity between latent constructs was not assessed using Fornell–Larcker criteria or HTMT ratios.

4. Results

The item-total correlation range includes an extremely low value (0.067), suggesting weak alignment with the overall construct. Retaining such an item without conducting sensitivity analyses or reporting alternative models is problematic.

Predictive validity was assessed using independent t-tests without controlling for potential confounders such as age, education, gender, or duration of treatment. Therefore, conclusions about predictive validity remain limited.

Convergent validity relied on a single-item self-rated health measure, which has inherent measurement limitations. This methodological constraint is not sufficiently acknowledged.

There are minor inconsistencies in reported degrees of freedom in some t-tests relative to group sample sizes, suggesting the need for statistical verification.

5. Discussion

The discussion occasionally implies causal interpretation of associations between health literacy and adherence, despite the cross-sectional design.

The comparison of the observed effect size (d=0.89) with meta-analytic estimates from other chronic diseases does not sufficiently account for differences in instruments, adherence measurement methods, and disease contexts. This comparison may overstate the strength of the findings.

The claim of superiority over other TB literacy tools based solely on S-CVI comparisons is not methodologically sound, as content validity indices are not directly comparable across studies with different expert panels and contexts.

Limitations are acknowledged but not deeply analyzed. In particular, the following require stronger emphasis:

Single-district validation,

Potential social desirability bias,

Lack of measurement invariance testing,

Absence of responsiveness analysis,

Small DR-TB subgroup size.

6. Structural and Reporting Issues

There are minor inconsistencies in statistical reporting (e.g., degrees of freedom) that require careful verification.

Frequent use of evaluative descriptors such as “excellent,” “robust,” and “strong” is not always anchored in clearly defined benchmarks.

Some adaptation decisions (e.g., replacing TB-specific wording with broader “health condition” terminology) may weaken the TB-specific focus of the instrument and require clearer theoretical justification.

**********

6. PLOS authors have the option to publish the peer review history of their article (what does this mean?). If published, this will include your full peer review and any attached files.

If you choose “no”, your identity will remain anonymous but your review may still be made public.

**Do you want your identity to be public for this peer review?**  For information about this choice, including consent withdrawal, please see our Privacy Policy.

Reviewer #1: **Yes:**  Dr. Parviz Shahmirzalou, PhD in Biostatistics.

Reviewer #2: No

**********

[NOTE: If reviewer comments were submitted as an attachment file, they will be attached to this email and accessible via the submission site. Please log into your account, locate the manuscript record, and check for the action link "View Attachments". If this link does not appear, there are no attachment files.]

To ensure your figures meet our technical requirements, please review our figure guidelines: https://journals.plos.org/plosone/s/figures

You may also use PLOS’s free figure tool, NAAS, to help you prepare publication quality figures: https://journals.plos.org/plosone/s/figures#loc-tools-for-figure-preparation.

NAAS will assess whether your figures meet our technical requirements by comparing each figure against our figure specifications.

# Author comment: Author response to Decision Letter 1

**DOI:** 10.1371/journal.pone.0352661.r002

7 May 2026

Journal requirements

1. Please ensure that your manuscript meets PLOS ONE's style requirements,

including those for file naming. The PLOS ONE style templates can be found at

https://journals.plos.org/plosone/s/file?id=wjVg/PLOSOne_formatting_sample_

main_body.pdf and https://journals.plos.org/plosone/s/file?id=ba62/PLOSOne_formatting_sample_title_

authors_affiliations.pdf

Thank you. We have revised the manuscript as per the journal requirements.

2. Your ethics statement should only appear in the Methods section of your manuscript. If your ethics statement is written in any section besides the Methods, please move it to the Methods section and delete it from any other section. Please ensure that your ethics statement is included in your manuscript, as the ethics statement entered into the online submission form will not be published alongside your manuscript. Addressed. Move to method section

The suggested changes can be found on page 18, line 373-379.

3. 3. Please ensure that you refer to Figure 1 in your text as, if accepted, production will need this reference to link the reader to the figure. Addressed. The suggested change can be found on page 214, line 11.

4. 4. Please include captions for your Supporting Information files at the end of your manuscript, and update any in-text citations to match accordingly. Please see our Supporting Information guidelines for more information: http://journals.plos.org/plosone/s/supporting-information. – we have removed supplementary files and have included only final validated tool. Addressed and included at the end of the manuscript. The suggested change can be found on page 46, line 785-787.

5. We note that there is identifying data in the Supporting Information file < Supplementary Table 2.docx >. Due to the inclusion of these potentially identifying data, we have removed this file from your file inventory. Prior to sharing human research participant data, authors should consult with an ethics committee to ensure data are shared in accordance with participant consent and all applicable local laws. Data sharing should never compromise participant privacy. It is therefore not appropriate to publicly share personally identifiable data on human research participants. The following are examples of data that should not be shared:

-Name, initials, physical address

-Ages more specific than whole numbers

-Internet protocol (IP) address

-Specific dates (birth dates, death dates, examination dates, etc.)

-Contact information such as phone number or email address

-Location data

-ID numbers that seem specific (long numbers, include initials, titled “Hospital ID”) rather than random (small numbers in numerical order)

Data that are not directly identifying may also be inappropriate to share, as in combination they can become identifying. For example, data collected from a small group of participants, vulnerable populations, or private groups should not be shared if they involve indirect identifiers (such as sex, ethnicity, location, etc.) that may risk the identification of study participants. Additional guidance on preparing raw data for publication can be found in our Data Policy (https://journals.plos.org/plosone/s/data-availability#loc-human-research-participant-data-and-other-sensitive-data) and in the following article: http://www.bmj.com/content/340/bmj.c181.long.

Please remove or anonymize all personal information (ID numbers that seem specific and Ages more specific than whole numbers), ensure that the data shared are in accordance with participant consent, and re-upload a fully anonymized data set. Please note that spreadsheet columns with personal information must be removed and not hidden as all hidden columns will appear in the published file. We have addressed this. Thank you

6. If the reviewer comments include a recommendation to cite specific previously published works, please review and evaluate these publications to determine whether they are relevant and should be cited. There is no requirement to cite these works unless the editor has indicated otherwise Thank you for this clarification. We confirm that no reviewer specifically recommended inclusion of additional citations or previously published works. Accordingly, no changes were made in this regard.

Reviewer Comments

1- "Study Participants": Describe participants (mean age (SD), working history, etc), if possible.

Thank you for this valuable suggestion. We have revised the “Study Participants” section to provide a clearer description of participant characteristics across all stages of adaptation and validation. Specifically, we have now included details on participants’ age ranges, years of professional experience for experts and healthcare providers, as well as demographic and clinical characteristics of TB patient participants, including mean age (SD), gender distribution, education status, comorbidity profile, and healthcare setting. In addition, we clarified that detailed characteristics of experts and healthcare providers are presented in S1 Table.

The suggested changes can be found on page 9-10, line 171-194.

2- It is better to add an explanation about the ratio of the sample size (393 participants). For example, point out the number of questions in HELIA.

Thank you for this important suggestion. We have now clarified the rationale for the validation sample size in the “Sample size and sampling” subsection. Specifically, we added that the HELIA-TB instrument contains 33 items, and the validation sample of 393 participants exceeded the commonly recommended ratio of 5–10 participants per item for psychometric validation and factor-analytic procedures. This strengthened the statistical power and adequacy of the validation analyses.

The suggested changes can be found on page 145, line 286-299

3- SPSS version 26 was used for quantitative analysis. Have you done CFA in SPSS?

If yes, explain in the manuscript or add the SPSS scripts to the supplementary materials.

(Usually, CFA HAVE DONE WITH AMOS, LISREL, or Smart-PLS).

Thank you for this observation. Confirmatory Factor Analysis (CFA) was conducted using the AMOS module available within the SPSS version 26 environment. We have now clarified this in the “Data Analysis” subsection of the manuscript by specifying the software and estimation method used for CFA.

The suggested change can be found on page 16, line 338-340.

4- If it's possible, rewrite and redesign Tables 1 and 2 to be small.

Thank you for this suggestion. To improve the readability and conciseness of the manuscript, we have moved Table 1 (detailed characteristics of experts and healthcare providers) to the supplementary materials as Supplementary S1 Table. Table 2 was retained in the main manuscript because the item-level content validity assessment is central to the psychometric validation process.

The suggested change can be found in S1 Table.

5- For the RMSEA index, report 95% Confidence Interval.

Thank you for this valuable suggestion. We agree that reporting the confidence interval for RMSEA enhances the interpretability and completeness of CFA reporting. Accordingly, the manuscript has been revised to include the RMSEA confidence interval.

The suggested change can be found on Page 34, line 540.

6- Check Link for Ref 14. I cannot access the link.

Thank you for highlighting this issue. We rechecked the link/reference for Ref. 14 and confirmed that it is accessible and functioning correctly from our side. Nevertheless, we have carefully verified the URL and reference formatting in the revised manuscript to ensure accessibility.

Reviewer #2

: 1. Title and Abstract

The title is structurally appropriate; however, the phrase “Cultural Adaptation and Validation” could be made more precise by indicating the type of validation conducted (e.g., confirmatory factor analysis, predictive validity), particularly since the manuscript reports CFA and multiple forms of construct validity. These important methodological components are not reflected in the title.

Thank you for this valuable suggestion. We agree that the title should better reflect the broader psychometric evaluation conducted in the study, including confirmatory factor analysis and construct validity assessments. Accordingly, we have revised the title to:

“Cultural Adaptation and Psychometric Validation of the Health Literacy Instrument for Adults with Tuberculosis (HELIA-TB) in India.”

In the abstract, the study is described as an “exploratory sequential mixed-methods design,” yet the operational linkage between the qualitative and quantitative phases is not clearly explained. It remains unclear how qualitative findings directly informed the quantitative validation or how integration occurred. Furthermore, the timeframe (March 2024–April 2025) raises concerns, as it overlaps with the manuscript submission period; clarification is required regarding whether data collection was fully completed prior to submission.

Thank you for this thoughtful observation. We have revised the abstract and Methods section to clarify the operational linkage between the qualitative and quantitative phases of the exploratory sequential mixed-methods design. Specifically, we now explain that findings from the qualitative phase, including expert review and cognitive interviews with TB patients and healthcare providers, directly informed item refinement, contextual adaptation, terminology simplification, and cultural modifications prior to quantitative psychometric validation. The revised instrument was subsequently evaluated through reliability and construct validity analyses in the larger quantitative sample.

The suggested change can be found on page 3, 52-60.

We also clarified the study timeline. Participant recruitment and data collection were completed prior to manuscript submission. The reported study period (March 2024–April 2025) reflects the overall duration of the study, including data cleaning, analysis, interpretation, and manuscript preparation following submission. We have now revised this to March 2024-March

The suggested change can be found on page 3, line 53-54.

The abstract reports effect sizes (Cohen’s d), but the scoring range and scaling method of the HELIA-TB are not specified, limiting interpretability of the magnitude of these effects. Additionally, the statement that no significant differences were observed by TB type or drug resistance status does not acknowledge the extremely small sample size in the DR-TB group (n=11), which substantially increases the risk of Type II error.

Thank you for these important observations. Additional clarification regarding HELIA-TB scoring has now been incorporated into the revised manuscript. Consistent with the original HELIA scoring protocol, raw scores were linearly transformed to a standardized 0–100 scale, with higher scores indicating higher health literacy levels, thereby improving interpretability of reported effect sizes.

The suggested change can be found on page 16-16, line 336-338.

We also acknowledge that the relatively small number of participants with drug-resistant TB (n = 11) substantially limited statistical power for subgroup comparisons and increased the possibility of Type II error. Accordingly, findings related to drug resistance status have been interpreted cautiously and this limitation has now been acknowledged in the manuscript.

The suggested change can be found on page 39-40, line 644-657.

The conclusion in the abstract states that the tool “has potential to enhance adherence,” which implies a causal relationship that cannot be supported by the cross-sectional design. This wording should be tempered.

Thank you for this important observation. The conclusion in the abstract has been revised to avoid implying a causal relationship between the instrument and treatment adherence. The wording has been tempered to emphasize the tool’s potential role in health literacy assessment and in informing targeted TB care interventions rather than directly enhancing adherence outcomes.

The suggested change can be found on page 4, line 76-78.

2. Introduction

The epidemiological statistics on TB incidence and mortality in India are presented without specifying the reference year, which is critical given annual variations in TB reporting.

Thank you for highlighting this. We have revised and now included the reference year. The suggested change can be found on page 5, line 85.

The assertion of a “growing consensus” favoring adaptation over new tool development is not adequately substantiated with comparative methodological or empirical evidence.

Thank you for this important observation. The wording has been revised to avoid overstating the level of consensus regarding adaptation approaches. The revised text now emphasizes that cultural adaptation and validation of established instruments is increasingly recognized as a practical and scientifically efficient strategy because it allows contextual relevance while maintaining conceptual comparability across settings and populations.

The suggested change can be found on page 6-7, line 126-129.

The manuscript states that HELIA was “developed in Iran in English,” which appears factually inaccurate. HELIA was originally developed in Persian and later translated into English. This point is important because it directly affects the justification of the translation and adaptation pathway.

Response: Thank you for this important observation. The manuscript has been clarified to better describe the adaptation pathway used in the present study. HELIA was originally developed in Iran, and the English version provided by the original developers was used as the reference version during the cultural adaptation process for the present study. This clarification has been incorporated to improve transparency regarding translation and adaptation procedures.

The suggested change can be found on Page 8, line 156-158.

The comparison with instruments such as HLQ and HLS-EU emphasizes licensing constraints, but the theoretical and structural differences between these tools and HELIA are insufficiently analyzed. The rationale for selecting HELIA appears more pragmatic (open access) than theoretically grounded.

Thank you for this important observation. The selection of HELIA was based not solely on considerations of accessibility, but on its conceptual structure, multidimensional framework, and contextual relevance for TB care within a lower-middle-income country setting. Unlike instruments such as REALM and TOFHLA, which primarily assess functional literacy skills, HELIA evaluates broader health literacy competencies including access to information, reading, understanding, appraisal, and decision-making, thereby aligning more closely with contemporary multidimensional health literacy frameworks and the practical self-management demands associated with TB treatment. Although HLQ and HLS-EU are comprehensive instruments with strong psychometric foundations, they were developed within substantially different sociocultural and health-system contexts and involve adaptation procedures that may include licensing restrictions, administrative permissions, and collaborative requirements that can limit independent contextual adaptation in resource-constrained settings. In contrast, HELIA was originally developed and validated in a lower-middle-income country context with healthcare and literacy realities more comparable to the present study population. Therefore, the rationale for selecting HELIA was both theoretically grounded and contextually informed, rather than based solely on pragmatic considerations.

3. Methods

Study Design

Although the study is described as an exploratory sequential mixed-methods design, the integration strategy is not adequately articulated. The manuscript does not explain how qualitative findings shaped item refinement in a traceable, systematic manner. No joint display or explicit integration framework is presented.

Thank you for this important observation. The exploratory sequential mixed-methods integration strategy was operationalized through iterative use of qualitative findings to guide refinement of the HELIA-TB instrument prior to quantitative psychometric validation.

Attachment Submitted filename: Response to reviewers.pdf

# Peer review: Decision Letter 1

**DOI:** 10.1371/journal.pone.0352661.r003

**Authors:** Muhammad Haroon Stanikzai, Muhammad Haroon Stanikzai

1 Jun 2026

PONE-D-26-06515R1

Cultural Adaptation and Psychometric Validation of the Health Literacy Instrument for Adults with Tuberculosis (HELIA-TB) in India

PLOS One

Dear Dr. Chauhan,

Thank you for submitting your manuscript to PLOS ONE. After careful consideration, we feel that it has merit but does not fully meet PLOS ONE’s publication criteria as it currently stands. Therefore, we invite you to submit a revised version of the manuscript that addresses the points raised during the review process.

Thank you for positively responding to reviewers' comments. The reviewers' still need some information before the manuscript can be accepted for publication.

Please submit your revised manuscript by Jul 16 2026 11:59PM. If you will need more time than this to complete your revisions, please reply to this message or contact the journal office at plosone@plos.org. When you're ready to submit your revision, log on to https://www.editorialmanager.com/pone/ and select the 'Submissions Needing Revision' folder to locate your manuscript file.

Please include the following items when submitting your revised manuscript:

- A letter that responds to each point raised by the academic editor and reviewer(s). You should upload this letter as a separate file labeled 'Response to Reviewers'.
- A marked-up copy of your manuscript that highlights changes made to the original version. You should upload this as a separate file labeled 'Revised Manuscript with Track Changes'.
- An unmarked version of your revised paper without tracked changes. You should upload this as a separate file labeled 'Manuscript'.

If you would like to make changes to your financial disclosure, please include your updated statement in your cover letter. Guidelines for resubmitting your figure files are available below the reviewer comments at the end of this letter.

If applicable, we recommend that you deposit your laboratory protocols in protocols.io to enhance the reproducibility of your results. Protocols.io assigns your protocol its own identifier (DOI) so that it can be cited independently in the future. For instructions see: https://journals.plos.org/plosone/s/submission-guidelines#loc-laboratory-protocols. Additionally, PLOS ONE offers an option for publishing peer-reviewed Lab Protocol articles, which describe protocols hosted on protocols.io. Read more information on sharing protocols at https://plos.org/protocols?utm_medium=editorial-email&utm_source=authorletters&utm_campaign=protocols.

As the corresponding author, your ORCID iD is verified in the submission system and will appear in the published article. PLOS supports the use of ORCID, and we encourage all coauthors to register for an ORCID iD and use it as well. Please encourage your coauthors to verify their ORCID iD within the submission system before final acceptance, as unverified ORCID iDs will not appear in the published article. *Only*  the individual author can complete the verification step; PLOS staff *cannot*  verify ORCID iDs on behalf of authors.

We look forward to receiving your revised manuscript.

Kind regards,

Muhammad Haroon Stanikzai

Academic Editor

PLOS One

Journal Requirements:

If the reviewer comments include a recommendation to cite specific previously published works, please review and evaluate these publications to determine whether they are relevant and should be cited. There is no requirement to cite these works unless the editor has indicated otherwise.

Please review your reference list to ensure that it is complete and correct. If you have cited papers that have been retracted, please include the rationale for doing so in the manuscript text, or remove these references and replace them with relevant current references. Any changes to the reference list should be mentioned in the rebuttal letter that accompanies your revised manuscript. If you need to cite a retracted article, indicate the article’s retracted status in the References list and also include a citation and full reference for the retraction notice.

**Additional Editor Comments:**

- Please arrange your in-text citations and references as per PLOS ONE Journal style.

- There is inconsistency in font size and style across the documment.

- Please carefully read your article for language and grammar correction.

[Note: HTML markup is below. Please do not edit.]

Reviewers' comments:

Reviewer's Responses to Questions

**Comments to the Author**

1. If the authors have adequately addressed your comments raised in a previous round of review and you feel that this manuscript is now acceptable for publication, you may indicate that here to bypass the “Comments to the Author” section, enter your conflict of interest statement in the “Confidential to Editor” section, and submit your "Accept" recommendation.

Reviewer #1: (No Response)

**********

2. Is the manuscript technically sound, and do the data support the conclusions?

The manuscript must describe a technically sound piece of scientific research with data that supports the conclusions. Experiments must have been conducted rigorously, with appropriate controls, replication, and sample sizes. The conclusions must be drawn appropriately based on the data presented.

Reviewer #1: Yes

**********

3. Has the statistical analysis been performed appropriately and rigorously?

Reviewer #1: Yes

**********

4. Have the authors made all data underlying the findings in their manuscript fully available?

The PLOS Data policy requires authors to make all data underlying the findings described in their manuscript fully available without restriction, with rare exception (please refer to the Data Availability Statement in the manuscript PDF file). The data should be provided as part of the manuscript or its supporting information, or deposited to a public repository. For example, in addition to summary statistics, the data points behind means, medians and variance measures should be available. If there are restrictions on publicly sharing data—e.g. participant privacy or use of data from a third party—those must be specified.

Reviewer #1: No

**********

5. Is the manuscript presented in an intelligible fashion and written in standard English?

PLOS ONE does not copyedit accepted manuscripts, so the language in submitted articles must be clear, correct, and unambiguous. Any typographical or grammatical errors should be corrected at revision, so please note any specific errors here.

Reviewer #1: Yes

**********

6. Review Comments to the Author

Please use the space provided to explain your answers to the questions above. You may also include additional comments for the author, including concerns about dual publication, research ethics, or publication ethics. (Please upload your review as an attachment if it exceeds 20,000 characters)

Reviewer #1: Dear Editor in Chief

After reviewing the authors’ responses, I would like to provide the following comments:

1- "Study Participants": Describe participants (mean age (SD), working history, etc), if possible.

This issue has been addressed. However, it would be preferable to present the information in a clearer table format. At minimum, reduce row lines to improve table readability.

2- It is better to add an explanation about the ratio of the sample size (393 participants).

This issue has been clarified.

3- SPSS version 26 was used for quantitative analysis. Have you done CFA in SPSS?

This issue has been addressed.

4- If it's possible, rewrite and redesign Tables 1 and 2 to be small.

This issue has been addressed.

5- For the RMSEA index, report 95% Confidence Interval.

This issue has been clarified.

6- Check Link for Ref 14. I cannot access the link

Reference 14 is currently presented as:

“14. Directorate of Census Operations, Gujarat”

References, especially reference 14, need to be checked again based on PloS ONE style.

**********

7. PLOS authors have the option to publish the peer review history of their article (what does this mean?). If published, this will include your full peer review and any attached files.

If you choose “no”, your identity will remain anonymous but your review may still be made public.

**Do you want your identity to be public for this peer review?**  For information about this choice, including consent withdrawal, please see our Privacy Policy.

Reviewer #1: **Yes:**  Parviz Shahmirzalou

**********

[NOTE: If reviewer comments were submitted as an attachment file, they will be attached to this email and accessible via the submission site. Please log into your account, locate the manuscript record, and check for the action link "View Attachments". If this link does not appear, there are no attachment files.]

To ensure your figures meet our technical requirements, please review our figure guidelines: https://journals.plos.org/plosone/s/figures

You may also use PLOS’s free figure tool, NAAS, to help you prepare publication quality figures: https://journals.plos.org/plosone/s/figures#loc-tools-for-figure-preparation.

NAAS will assess whether your figures meet our technical requirements by comparing each figure against our figure specifications.

Attachment Submitted filename: Response to Editor.docx

# Author comment: Author response to Decision Letter 2

**DOI:** 10.1371/journal.pone.0352661.r004

2 Jun 2026

Dear Editor in Chief

Thank you for providing opportunity to address the comments. Please find the response beneath each point.

1- "Study Participants": Describe participants (mean age (SD), working history, etc), if possible.

This issue has been addressed. However, it would be preferable to present the information in a clearer table format. At minimum, reduce row lines to improve table readability.

Response:

Thank you for this suggestion. We have revised the participant characteristics table to improve readability by summarizing participant demographics by stakeholder group and reducing excessive row lines.

2- It is better to add an explanation about the ratio of the sample size (393 participants).

This issue has been clarified.

Response: Thank you

3- SPSS version 26 was used for quantitative analysis. Have you done CFA in SPSS?

This issue has been addressed.

Response: Thank you

4- If it's possible, rewrite and redesign Tables 1 and 2 to be small.

This issue has been addressed.

Response: Thank you

5- For the RMSEA index, report 95% Confidence Interval.

This issue has been clarified.

Response: Thank you

6- Check Link for Ref 14. I cannot access the link

Reference 14 is currently presented as:

“14. Directorate of Census Operations, Gujarat”

References, especially reference 14, need to be checked again based on PloS ONE style.

Response: Thank you. Reference 14 has been updated and revised as per PloS ONE style.

Attachment Submitted filename: Response to reviewer.docx

# Editor report: Decision Letter 2

**DOI:** 10.1371/journal.pone.0352661.r005

**Authors:** Muhammad Haroon Stanikzai, Muhammad Haroon Stanikzai, Muhammad Haroon Stanikzai

8 Jun 2026

PONE-D-26-06515R2

Cultural Adaptation and Psychometric Validation of the Health Literacy Instrument for Adults with Tuberculosis (HELIA-TB) in India

PLOS One

Dear Dr. Chauhan,

Thank you for submitting your manuscript to PLOS ONE. After careful consideration, we feel that it has merit but does not fully meet PLOS ONE’s publication criteria as it currently stands. Therefore, we invite you to submit a revised version of the manuscript that addresses the points raised during the review process.

Thank you for addressing initial comments raised by esteemed reviewers. Before acceptance, some further editorial corrections are needed to be provided, detailed at additional editor comments section.

Please submit your revised manuscript by Jul 23 2026 11:59PM. If you will need more time than this to complete your revisions, please reply to this message or contact the journal office at plosone@plos.org. When you're ready to submit your revision, log on to https://www.editorialmanager.com/pone/ and select the 'Submissions Needing Revision' folder to locate your manuscript file.

Please include the following items when submitting your revised manuscript:

- A letter that responds to each point raised by the academic editor and reviewer(s). You should upload this letter as a separate file labeled 'Response to Reviewers'.
- A marked-up copy of your manuscript that highlights changes made to the original version. You should upload this as a separate file labeled 'Revised Manuscript with Track Changes'.
- An unmarked version of your revised paper without tracked changes. You should upload this as a separate file labeled 'Manuscript'.

If you would like to make changes to your financial disclosure, please include your updated statement in your cover letter. Guidelines for resubmitting your figure files are available below the reviewer comments at the end of this letter.

If applicable, we recommend that you deposit your laboratory protocols in protocols.io to enhance the reproducibility of your results. Protocols.io assigns your protocol its own identifier (DOI) so that it can be cited independently in the future. For instructions see: https://journals.plos.org/plosone/s/submission-guidelines#loc-laboratory-protocols. Additionally, PLOS ONE offers an option for publishing peer-reviewed Lab Protocol articles, which describe protocols hosted on protocols.io. Read more information on sharing protocols at https://plos.org/protocols?utm_medium=editorial-email&utm_source=authorletters&utm_campaign=protocols.

As the corresponding author, your ORCID iD is verified in the submission system and will appear in the published article. PLOS supports the use of ORCID, and we encourage all coauthors to register for an ORCID iD and use it as well. Please encourage your coauthors to verify their ORCID iD within the submission system before final acceptance, as unverified ORCID iDs will not appear in the published article. *Only*  the individual author can complete the verification step; PLOS staff *cannot*  verify ORCID iDs on behalf of authors.

We look forward to receiving your revised manuscript.

Kind regards,

Muhammad Haroon Stanikzai

Academic Editor

PLOS One

Journal Requirements:

If the reviewer comments include a recommendation to cite specific previously published works, please review and evaluate these publications to determine whether they are relevant and should be cited. There is no requirement to cite these works unless the editor has indicated otherwise.

Please review your reference list to ensure that it is complete and correct. If you have cited papers that have been retracted, please include the rationale for doing so in the manuscript text, or remove these references and replace them with relevant current references. Any changes to the reference list should be mentioned in the rebuttal letter that accompanies your revised manuscript. If you need to cite a retracted article, indicate the article’s retracted status in the References list and also include a citation and full reference for the retraction notice.

**Additional Editor Comments:**

- Please look at PLOS ONE guidelines for in-text citations and references and correct them accordingly (You can also review papers at PLOS ONE).

- Several terms were used with its full form in the manuscript several times. Once a term is abbreviated for the first time, it is not necessary to use the abbreviated form with its full form repeatedly. Only the abbreviated terms can be used subsequently across the document.

- Please proofread the entire article for language and grammar correction (line 222; there is no need for " sign). Please also review tables and figures.

[Note: HTML markup is below. Please do not edit.]

[NOTE: If reviewer comments were submitted as an attachment file, they will be attached to this email and accessible via the submission site. Please log into your account, locate the manuscript record, and check for the action link "View Attachments". If this link does not appear, there are no attachment files.]

To ensure your figures meet our technical requirements, please review our figure guidelines: https://journals.plos.org/plosone/s/figures

You may also use PLOS’s free figure tool, NAAS, to help you prepare publication quality figures: https://journals.plos.org/plosone/s/figures#loc-tools-for-figure-preparation.

NAAS will assess whether your figures meet our technical requirements by comparing each figure against our figure specifications.

# Author comment: Author response to Decision Letter 3

**DOI:** 10.1371/journal.pone.0352661.r006

11 Jun 2026

Thank you for your careful review and constructive suggestions.

We have revised the manuscript in accordance with the recommendations. Specifically:

1. PLOS ONE citation and reference style: All in-text citations and references were reviewed and reformatted to align with PLOS ONE guidelines. Reference formatting, author listings, journal abbreviations, punctuation, and web-based citations were checked and corrected where necessary.

2. Use of abbreviations: Repeated use of terms with both full forms and abbreviations has been corrected. Terms are now defined at first mention and abbreviated forms are used consistently throughout the remainder of the manuscript.

3. Language and grammar: The manuscript has undergone a comprehensive proofreading for grammar, punctuation, consistency, and readability. The unnecessary quotation marks identified by the reviewer have been removed, and several minor language edits have been made throughout the text.

4. Tables and figures: All tables and figures were reviewed for consistency, formatting, clarity, and alignment with journal requirements. Minor formatting corrections were implemented where needed.

Attachment Submitted filename: REsponse to reviewer_R2.docx

# Editor report: Decision Letter 3

**DOI:** 10.1371/journal.pone.0352661.r007

**Authors:** Muhammad Haroon Stanikzai, Muhammad Haroon Stanikzai, Muhammad Haroon Stanikzai, Muhammad Haroon Stanikzai

14 Jun 2026

Cultural Adaptation and Psychometric Validation of the Health Literacy Instrument for Adults with Tuberculosis (HELIA-TB) in India

PONE-D-26-06515R3

Dear Dr. Chauhan,

We’re pleased to inform you that your manuscript has been judged scientifically suitable for publication and will be formally accepted for publication once it meets all outstanding technical requirements.

Within one week, you’ll receive an e-mail detailing the required amendments. When these have been addressed, you’ll receive a formal acceptance letter and your manuscript will be scheduled for publication.

An invoice will be generated when your article is formally accepted. Please note, if your institution has a publishing partnership with PLOS and your article meets the relevant criteria, all or part of your publication costs will be covered. Please make sure your user information is up-to-date by logging into Editorial Manager at Editorial Manager® and clicking the ‘Update My Information' link at the top of the page. For questions related to billing, please contact billing support.

If your institution or institutions have a press office, please notify them about your upcoming paper to help maximize its impact. If they’ll be preparing press materials, please inform our press team as soon as possible -- no later than 48 hours after receiving the formal acceptance. Your manuscript will remain under strict press embargo until 2 pm Eastern Time on the date of publication. For more information, please contact onepress@plos.org.

Kind regards,

Muhammad Haroon Stanikzai

Academic Editor

PLOS One

Additional Editor Comments (optional):

Please carefully review the Proof for any typos and language corrections.

Reviewers' comments:

# Editor report: Acceptance letter

**DOI:** 10.1371/journal.pone.0352661.r008

**Authors:** Muhammad Haroon Stanikzai, Muhammad Haroon Stanikzai, Muhammad Haroon Stanikzai, Muhammad Haroon Stanikzai

PONE-D-26-06515R3

PLOS One

Dear Dr. Chauhan,

I'm pleased to inform you that your manuscript has been deemed suitable for publication in PLOS One. Congratulations! Your manuscript is now being handed over to our production team.

At this stage, our production department will prepare your paper for publication. This includes ensuring the following:

* All references, tables, and figures are properly cited

* All relevant supporting information is included in the manuscript submission,

* There are no issues that prevent the paper from being properly typeset

You will receive further instructions from the production team, including instructions on how to review your proof when it is ready. Please keep in mind that we are working through a large volume of accepted articles, so please give us a few days to review your paper and let you know the next and final steps.

Lastly, if your institution or institutions have a press office, please let them know about your upcoming paper now to help maximize its impact. If they'll be preparing press materials, please inform our press team within the next 48 hours. Your manuscript will remain under strict press embargo until 2 pm Eastern Time on the date of publication. For more information, please contact onepress@plos.org.

You will receive an invoice from PLOS for your publication fee after your manuscript has reached the completed accept phase. If you receive an email requesting payment before acceptance or for any other service, this may be a phishing scheme. Learn how to identify phishing emails and protect your accounts at https://explore.plos.org/phishing.

If we can help with anything else, please email us at customercare@plos.org.

Thank you for submitting your work to PLOS ONE and supporting open access.

Kind regards,

PLOS ONE Editorial Office Staff

on behalf of

Dr. Muhammad Haroon Stanikzai

Academic Editor

PLOS One
